# Synthesis, Anticancer Evaluation, mRNA Expression Analysis, and Molecular Docking of Fluorinated Picolinamide–Benzothiazole Derivatives Against Breast Cancer‐Related Genes CDK2, ERK2, and BCL2

**DOI:** 10.1002/jbt.71025

**Published:** 2026-08-17

**Authors:** Nagalakshmamma Vadabingi, Venkataswamy Mallepogu, Surendra Pothuraju, Chiranjeevi Pasala, Sumithra Poreddy, Uma Maheswari Amineni, Suresh Reddy Cirandur, Balaji Meriga

**Affiliations:** ^1^ Department of Chemistry Sri Venkateswara University Tirupati India; ^2^ Department of Biochemistry Sri Venkateswara University Tirupati India; ^3^ Department of Chemistry Vellore Institute of Technology (VIT) Vellore India; ^4^ Bioinformatics Centre, Department of Bioinformatics SVIMS University Tirupati India

**Keywords:** antioxidant activity, benzothiazole, breast cancer, molecular docking, molecular dynamics simulation, picolinamide derivatives

## Abstract

The present study focuses on the design, synthesis, characterization, and biological evaluation of two new series of derivatives 4‐(4‐amino‐3‐fluorophenoxy)‐N‐methylpicolinamide (**3a–e**) and 2‐aminobenzothiazole (**5a‐e**) as potential antioxidant and anticancer agents. The compounds were synthesized via a one‐pot condensation reaction of 4‐(4‐amino‐3‐fluorophenoxy)‐N‐methylpicolinamide and 2‐aminobenzothiazole with various phenyl isocyanates, isothiocyanates, and sulfonamides in the presence of triethylamine at 60°C. Reaction progress was monitored by TLC, and products were purified using column chromatography. Structural elucidation was achieved using FT‐IR, ^1^H NMR, ^13^C NMR, HRMS, and CHN analyses. Antioxidant activity, evaluated by DPPH and ABTS assays, revealed that compounds **3d** and **5b** exhibited the highest radical scavenging potential, with IC_50_ values of 21.45 μg/mL and 20.05 μg/mL (DPPH) and 22.24 μg/mL and 21.98 μg/mL (ABTS), comparable to ascorbic acid. Cytotoxicity assessment by MTT assay demonstrated strong antiproliferative activity of **3d** and **5b** against MDA‐MB‐231 breast cancer cells (IC_50_ = 22.13 μg/mL and 20.11 μg/mL), with minimal toxicity toward normal 3T3‐L1 cells. Fluorescence microscopy and flow cytometry confirmed apoptosis profile induction and G2/M phase arrest. The qRT‐PCR analysis showed significant down‐regulation of Bcl‐2, CDK2, and ERK2 gene expression, correlating with apoptotic activation. Molecular docking and MM‐GBSA analyses revealed stable and high‐affinity binding of **3d** and **5b** with Bcl‐2, CDK2, and ERK2 proteins, supported by multiple hydrogen bonding and π–π interactions. Molecular dynamics simulations (150 ns) confirmed the structural stability of these complexes. Overall, compounds **3d** and **5b** emerged as promising dual‐function ‘antioxidant and anticancer’ candidates and in vivo evaluations for therapeutic development.

## Graphical abstract

The graphical abstract summarizes the synthesis of novel benzothiazole‐linked derivatives and their biological evaluation. The compounds exhibited significant antioxidant activity, notable cytotoxic effects against MDA‐MB‐231 cells, apoptosis induction with G2/M arrest, gene regulation, and favorable molecular docking interactions, supporting their therapeutic potential.

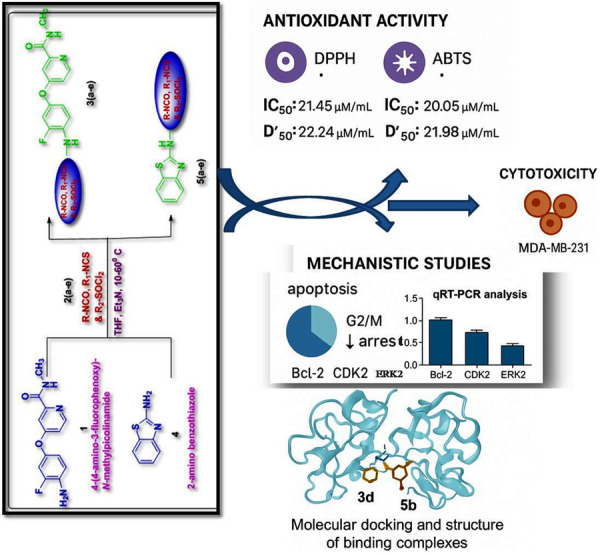

## 1 Introduction

Breast cancer is a globally prevalent disease that affects both men and women; however, it is significantly more prevalent in women [1]. The World Health Organization (WHO) anticipates a 70% increase in the number of new cases over the next two decades, with an estimated 2.3 million new cases diagnosed in 2020 [2]. In India, breast cancer is increasingly prevalent among women. In 2020, the Indian Council of Medical Research (ICMR) reported that there were more than 1.6 lakh new cases of breast cancer, a substantial increase from previous years. Increased awareness and detection technologies, delayed childbearing, lifestyle modifications, and hormone prescriptions have all contributed to the increase in diagnoses. The prevalence of breast cancer in India has been increasing, particularly in urban areas where lifestyle changes, such as late marriages, increased alcohol consumption, and a dearth of physical activity, are becoming more prevalent [3]. The majority of cancer treatments that are currently available, including radiation and chemotherapeutic medications, have only temporary therapeutic benefits and are constrained by a small therapeutic index, significant adverse effects, and frequently established resistance [4]. Consequently, there is an increasing necessity to identify and create innovative treatment strategies, particularly new chemotherapeutics with decreased adverse effects and increased efficacy.

In recent years, synthetic drug therapies for breast cancer have undergone a significant improvement, offering more personalized, effective, and focused therapeutic alternatives [5]. The treatment option is determined by a variety of factors, such as the patient's overall health, the molecular characteristics of the tumor, and the stage of the cancer. These synthetic medications have the potential to significantly improve the quality of life and survival rates of breast cancer patients when administered correctly.

Researchers have been intrigued by benzothiazole derivatives as potential cancer treatments due to their capacity to alter critical cellular pathways [6, 7, 8]. Key proteins and kinases, including CDK2, ERK2, and BCL2, are essential for the proliferation, survival, apoptosis and cell cycle of cancer cells [9, 10, 11]. CDK2 is a critical enzyme that regulates the progression of the cell cycle, with a particular emphasis on the transition from G1 to S phase. Uncontrolled cell proliferation, which is a hallmark of malignancy, may result from CDK2 dysregulation. In numerous malignancies, including breast cancer, CDK2 is frequently overexpressed or hyperactivated. Numerous investigations have demonstrated that benzothiazole compounds may function as potent CDK2 inhibitors. By either interfering with the interaction of CDK2 with cyclins or connecting to the ATP‐binding site, these medications have the potential to effectively block the cell cycle [12]. This obstructs the progression of cancer cells through critical phases of cell division, resulting in cell cycle arrest and diminished cancer cell development. Benzothiazole drugs that inhibit CDK2 may induce G1/S phase arrest, thereby preventing cancer cells from proliferating and dividing. This effect is especially advantageous in cancer therapy, as it diminishes the proliferation of cancer cells. The mitogen‐activated protein kinase (MAPK) signaling system, which regulates critical processes such as cell proliferation, survival, differentiation, and apoptosis, includes extracellular signal‐regulated kinase 2 (ERK2). The MAPK pathway, particularly the ERK pathway, is typically overactivated in cancer cells, which facilitates tumor development and metastasis. It has been established that benzothiazole derivatives interact with critical components of the MAPK/ERK signaling cascade, thereby preventing the phosphorylation of ERK2. These drugs may disrupt downstream signaling pathways that regulate cell survival and proliferation by inhibiting ERK2 activation [13].

The risk of metastasis may be reduced by benzothiazole compounds that inhibit ERK2 signaling, which in turn reduces the ability of cancer cells to invade and migrate. Consequently, these compounds are highly effective in reducing the rate of cancer progression, particularly in aggressive breast cancer subtypes that frequently involve the deregulation of the MAPK pathway.

BCL2 (B‐cell lymphoma 2) is essential for the survival of cells and the prevention of apoptosis. It prevents programmed cell death by inhibiting the activity of pro‐apoptotic proteins, including BAX and Bak [14]. The ability of cancer cells to circumvent apoptosis is typically linked to the overexpression of BCL2, which results in tumor growth, chemo resistance, and metastasis. The expression of anti‐apoptotic proteins, such as BCL2, may be influenced by benzothiazole derivatives. Several studies have shown that the expression of BCL2 may be reduced by these compounds, which can lead to apoptosis in cancer cells. Benzothiazole derivatives, when combined with other chemotherapeutic or targeted regimens, increase the susceptibility of cancer cells to death signals by decreasing BCL2 levels. The efficacy of benzothiazole derivatives in modulating BCL2 may be influenced by a shift in the equilibrium between pro‐ and anti‐apoptotic proteins, such as an increase in BAX activity and a decrease in BCL2 activity. This modification has the potential to induce apoptosis in cancer cells, which would impair their ability to thrive in conditions that would typically facilitate their growth.

The current aim is to create novel hybrid anti‐cancer pharmaceuticals that improve therapeutic safety profiles and mitigate potential treatment resistance by modulating multiple targets and reducing toxicity.

## 2 Materials and Methods

### 2.1 Chemicals and Instruments

All synthetic reactions were carried out in oven‐dried glassware. Moisture‐ and air‐sensitive transformations were conducted under an inert argon atmosphere. Unless otherwise stated, all reagents and solvents were procured from commercial suppliers and used without further purification. Melting points were measured using a Boethius melting point apparatus and are reported without correction. Nuclear magnetic resonance (NMR) spectra were recorded on a Varian Gemini 400 MHz spectrometer. Chemical shifts were referenced to residual solvent signals: chloroform‐d (δH 7.26 ppm, δC 77.16 ppm) and DMSO‐d6 (δH 2.50 ppm, δC 39.53 ppm) for ^1^H and ^13^C NMR (100 MHz), respectively. All chemical shift values are expressed in parts per million (δ, ppm). Mass spectrometric analyses were performed using an AMD 604 spectrometer, while high‐resolution mass spectra (HRMS) were acquired via electrospray ionization (ESI).

### 2.2 General Synthesis of Compounds 3(a–e) and 5(a–e)

All reagents and solvents were of analytical grade and used without further purification. Reactions were carried out under a dry nitrogen atmosphere where required. Thin‐layer chromatography (TLC) on silica gel plates was used to monitor reaction progress. Melting points were determined in open capillaries and are uncorrected.

### 2.3 Synthesis of Substituted Urea/Thiourea Derivatives 3(a–e)

A solution of 4‐(4‐amino‐3‐fluorophenoxy)‐N‐methylpicolinamide (1) (1.0 equiv) was prepared in dry THF (20 mL) and cooled to 10°C. To this, triethylamine (Et_3_N, 1.5 equiv) was added dropwise under stirring. The appropriate aryl isocyanate (R–NCO) or aryl isothiocyanate (R_1_–NCS) (1.1 equiv) was then added slowly, and the reaction mixture was stirred at 10°C–60°C for 4–6 h. After completion (TLC monitoring), the reaction mixture was poured into ice‐cold water and extracted with ethyl acetate (3 × 25 mL). The combined organic layers were washed with brine, dried over anhydrous sodium sulfate, and concentrated under reduced pressure. The crude product was purified by recrystallization from ethanol to afford the target compounds (**3a–e**) in good yields.

### 2.4 Synthesis of Benzothiazole‐Linked Derivatives 5(a–e)

**Scheme 1:**
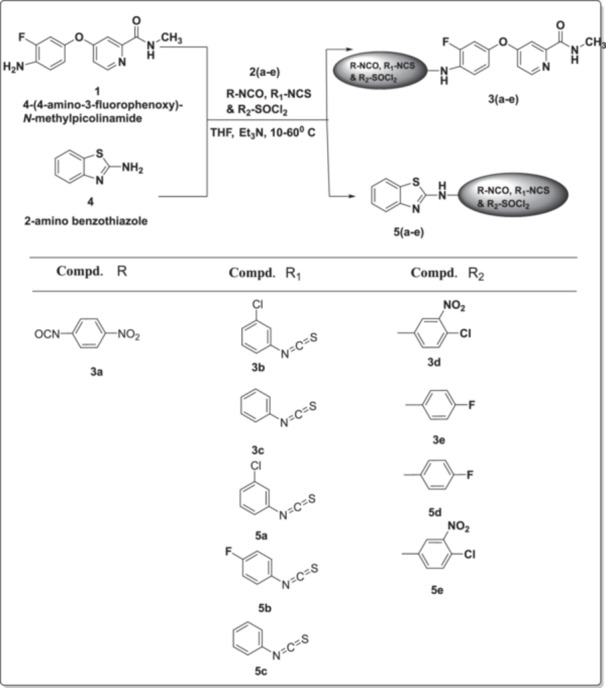
Synthesis of 4‐(4‐amino‐3‐fluorophenoxy)‐N‐methyl picolinamide and 2‐Aminobenzothiazole derivatives of **3a‐e** and **5a‐e**.

A similar procedure was followed for the synthesis of compounds 5(a–e) using 2‐aminobenzothiazole (4) (1.0 equiv) as the nucleophilic substrate. Compound 4 was dissolved in dry THF (20 mL), cooled to 10°C, and treated with Et_3_N (1.5 equiv). The corresponding aryl isocyanate, aryl isothiocyanate, or substituted sulfonyl chloride (R_2_–SOCl_2_) (1.1 equiv) was added portion‐wise with continuous stirring. The reaction mixture was maintained at 10°C–60°C for 5–7 h. Upon completion, the mixture was quenched with cold water, and the precipitated solid was filtered, washed with water, and dried. Further purification was achieved by recrystallization from ethanol to yield pure benzothiazole‐based derivatives (**5a–e**). The synthesized compounds were characterized by FT‐IR, ^1^H NMR, ^13^C NMR, Elemental analysis, and mass spectrometry, confirming the formation of urea/thiourea and sulfonamide linkages as depicted in Scheme 1.

### 2.5 Spectral Data

#### 2.5.1 4‐(3‐fluoro‐4‐(3‐(3‐nitrophenyl)ureido)phenoxy)‐N‐methylpicolinamide (3a)

Chemical Formula: C_20_H_16_FN_5_O_5_; Yellow solid, m.p. 230‐235; Yield 80%; IR (KBr) (ν_max_/cm^‐1^): 688, 949, 1022, 1218, 1308, 1350, 1425, 1525, 1661, 2998, 3448; ^1^H NMR (400 MHz, DMSO‐*d*_6_, ppm): *δ* 9.57 (s, 1H, NH), 8.30(s, 1H, Ar‐H), 8.28 (s, 1H, Ar‐H), 8.27‐26 (d, 1H, Ar‐H), 7.95 (d, 1H, Ar‐H), 7.93 (d, 1H, Ar‐H), 7.57‐7.56 (d, 1H, Ar‐H), 7.52‐7.49 (d, 1H, Ar‐H), 7.22 (s, 1H, Ar‐H), 6.87‐7.86 (q, 1H, NH–CH_3_) 6.77 (s, 1H, NH), 6.72 (d, 1H, Ar‐H), 6.70‐6.69 (d, 1H, Ar‐H), 1.97 (d, 3H, ‐CH_3_; ^13^C NMR (100 MHz, DMSO‐*d*_6_): *δ* 26.3, 117.2, 119.0, 120.0, 120.1, 122.0, 122.6, 122.8, 124.7, 129.8, 129.9, 130.2, 134.2, 150.0, 151.6, 153.1, 164.9, 179.9; HRMS (m/z): 425.03 Calcd. For C_20_H_16_FN_5_O_5_found 426 (M + H)^+^; Elemental Analysis: C 56.47, H 9.79, N 16.46, S 10.81 found C 48.47, H 4.41, N 9.58, S 8.80.


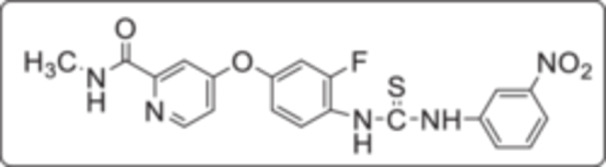


#### 2.5.2 4‐(4‐(3‐(3‐chlorophenyl)thioureido)‐3‐fluorophenoxy)‐N‐methylpicolinamide (3b)

Chemical Formula: C_20_H_16_ClFN_4_O_2_ S; White solid, m.p. 225‐228; Yield 89%; IR (KBr) (ν_max_/cm^‐1^): 698, 948, 1021, 1308, 1422, 1661, 2998, 3438; ^1^H NMR (400 MHz, DMSO‐*d*_6_, ppm): *δ* 8.84 (s, 1H, NH), 8.63 (s, 1H, Ar‐H), 8.54 (s, 1H, NH), 8.35‐8.30 (q, 1H, NH‐CH_3_), 7.43‐7.41 (d, 1H, Ar‐H), 7.27‐25 (d, 1H, Ar‐H), 7.19 (s, 1H, Ar‐H), 7.14‐12 (m, 1H, Ar‐H), 7.07‐7.04 (d, 1H, Ar‐H), 6.98‐6.96 (d, 1H, Ar‐H), 6.90‐6.87 (d,1H, Ar‐H), 6.83‐6.81(d,1H, Ar‐H), 6.85(s,1H, Ar‐H), 2.00 (d, 3H, ‐CH_3_; ^13^C NMR (100 MHz, DMSO‐*d*_6_): *δ* 26.3, 117.2, 119.0, 120.0, 120.1, 122.0, 122.6, 124.6, 126.0, 129.8, 130.2, 134.2, 140.3, 150.0, 151.6, 153.1, 164.7, 165.7, 171.9, 179.9: HRMS (m/z): 430.09 Calcd. For C_20_H_16_ClFN_4_O_2_ S found 431 (M + H)^+^; Elemental Analysis: C 55.76, H 3.79, N 13.46, S 7.81 found C 42.47, H 2.41, N 11.58, S 6.80.


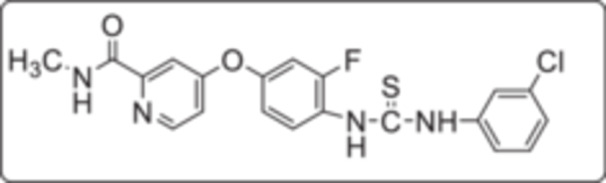


#### 2.5.3 4‐(3‐fluoro‐4‐(3‐phenylthioureido)phenoxy)‐N‐methylpicolinamide (3c)

Chemical Formula: C_20_H_17_FN_4_O_2_S; White solid, m.p. 230‐233; Yield 88%; IR (KBr) (ν_max_/cm^‐1^): 698, 947, 1024, 1308, 1423, 1663, 2997, 3444; ^1^H NMR (400 MHz, DMSO‐*d*_6_, ppm): *δ* 8.84 (s, 1H, NH), 8.62 (s, 1H, Ar‐H), 8.35‐30 (q, 1H, NH‐CH_3_), 8.07‐8.06 (d, 1H, Ar‐H), 7.49‐7.46 (m, 1H, NH), 7.26‐7.24 (d, 1H, Ar‐H), 7.10 (s,1H, Ar‐H), 7.00‐6.96 (dd, 1H, Ar‐H), 6.90‐6.87 (d, 1H, Ar‐H), 6.85 (s,1H, NH), 6.83‐6.81 (d,1H, Ar‐H), 2.87 (d, 3H, ‐CH_3_; ^13^C NMR (100 MHz, DMSO‐*d*_6_): *δ* 26.2, 106.7, 108.9, 110.3, 114.7, 116.2, 119.5, 119.9, 125.2, 125.3, 2126.9, 127.3, 150.0, 152.2, 164.5, 165.6, 180.4; HRMS (m/z): 396.11 Calcd. For C_20_H_17_FN_4_O_2_ S found 397.09 (M + H)^+^; Elemental Analysis: C 60.59, H 4.32, N 14.13, S 8.09 found C 60.00, H 3.32, N 12.10, S 7.19.


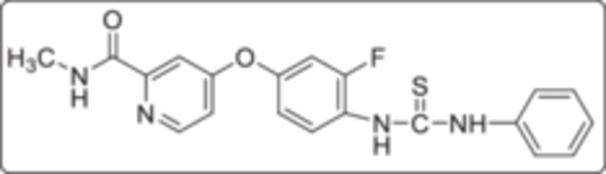


#### 2.5.4 4‐(4‐((4‐chloro‐3‐nitrophenyl)sulfonamido)‐3‐fluorophenoxy)‐N Methylpicolinamide (3 d)

Chemical Formula: C_19_H_14_ClFN_4_O_6_S; White solid, m.p. 236‐239; Yield 90%; IR (KBr) (ν_max_/cm^‐1^): 698, 1028, 1308, 1424, 1662, 2997, 3452; ^1^H NMR (400 MHz, DMSO‐*d*_6_, ppm):*δ* 8.84 (s, 1H, NH), 8.62 (s, 1H, Ar‐H), 8.35‐8.30 (m, 3H, Ar‐H), 8.13 (s, 1H, Ar‐H), 8.07‐8.06 (d, 1H, Ar‐H), 7.49‐7.46 (m, 1H, NH), 7.19 (s, 1H, Ar‐H), 6.90‐6.87 (d, 1H, Ar‐H), 6.83‐6.81 (d, 1H, Ar‐H), 2.91‐2.90 (d, 3H, CH_3_); ^13^C NMR (100 MHz, DMSO‐*d*_6_): *δ*26.1, 108.0, 109.0, 113.8, 115.0, 123.4, 125.6, 128.2, 130.7, 131.9, 133.8, 135.6, 145.3, 147.2, 149.6, 150.0, 155.0, 160.0, 166.7; HRMS (m/z): 480.03 Calcd. For C_19_H_14_ClFN_4_O_6_S found 481 (M + H)^+^; Elemental Analysis: C 49.46, H 9.43, N 11.65, S 8.67; found C 48.47, H 9.41, N 9.58, S 8.60.


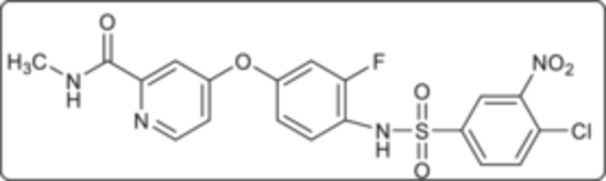


#### 2.5.5 4‐(3‐fluoro‐4‐((4‐fluorophenyl)sulfonamido)phenoxy)‐N‐methylpicolinamide (3e)

Chemical Formula: C_19_H_15_F_2_N_3_O_4_S; White solid, m.p. 232‐235; Yield 90%; IR (KBr) (ν_max_/cm^‐1^): 695, 843, 948, 1021, 1211, 1309, 1424, 1659, 2996, 3443; ^1^H NMR (400 MHz, DMSO‐*d*_6_, ppm): *δ* 8.72‐71 (d, 1H, Ar‐H), 8.62‐8.61 (s, 1H, Ar‐H), 7.80‐7.78 (m, 4H, Ar‐H), 7.78 (s, 1H, Ar‐H), 7.64‐7.60 (q,1H, NH), 7.28 (s, 1H, NH), 7.01‐7.00 (d, 1H, Ar‐H), 6.99 (d, 1H, Ar‐H), 6.96 (s, 1H, Ar‐H), 1.96 (d, 3H, CH_3_); ^13^C NMR (100 MHz, DMSO‐*d*_6_): *δ* 21.0, 114.9, 115.3, 116.1, 116.33, 116.39, 116.6, 118.0, 128.1, 128.2, 129.4, 129.5, 131.5, 131.6, 141.3, 162.1, 164.5; HRMS (m/z): 419.01 Calcd. For C_19_H_15_F_2_N_3_O_4_S found 420.09 (M + H)^+^; Elemental Analysis: C 54.41, H 3.61, N 10.02, S 7.64 found C 52.11, H 3.01, N 8.02, S 6.54.


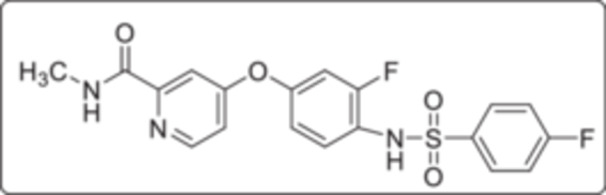


#### 2.5.6 1‐(benzo[d]thiazol‐2‐yl)‐3‐(3‐chlorophenyl)thiourea (5a)

Chemical Formula: C_14_H_10_ClN_3_S_2_; White solid, m.p. 160‐163; Yield 92%; IR (KBr) (ν_max_/cm^‐1^): 546, 657, 699, 769, 948, 1020, 1219, 1351, 1429, 1536, 1652, 2996, 3447; ^1^H NMR (400 MHz, DMSO‐*d*_6_, ppm): *δ* 11.31 (s, 1H, NH), 10.30 (s, 1H, NH), 8.27 (d, 1H, Ar‐H), 7.96 (s, 1H, Ar‐H), 7.94 (d, 1H, Ar‐H), 7.97‐96 (d, 1H, Ar‐H), 7.53‐52 (d, 1H, Ar‐H), 7.51‐50 (d, 1H, Ar‐H), 7.23‐7.21 (d, 1H, Ar‐H), 7.07‐7.06 (d, 1H, Ar‐H); ^13^C NMR (100 MHz, DMSO‐*d*_6_): 117.1, 121.2, 122.8, 123.4, 126.4, 128.0, 128.7, 130.7, 131.9, 145.4, 147.2, 153.2, 164.0, 167.8; HRMS (m/z): 319.00 Calcd. For C_14_H_10_ClN_3_S_2_ found 420.09 (M + H)^+^; Elemental Analysis: C 52.58, H 3.15, N 13.14, S 20.05 found C 51.48, H 3.05, N 12.04, S 19.00.


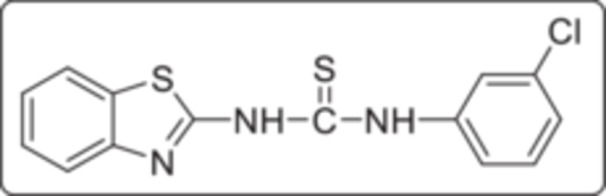


#### 2.5.7 1‐(benzo[d]thiazol‐2‐yl)‐3‐(4‐fluorophenyl)thiourea (5b)

Chemical Formula: C_14_H_10_FN_3_S_2_; White solid, m.p. 162‐165; Yield 92%; IR (KBr) (ν_max_/cm^‐1^): 698, 947, 1023, 1309, 1424, 1663, 2997, 3441; ^1^H NMR (400 MHz, DMSO‐*d*_6_, ppm): *δ* 11.60 (s, 1H, NH), 10.13(s, 1H, NH), 8.28‐8.26(d, 1H, Ar‐H), 7.52‐7.47 (d, 1H, Ar‐H), 7.45‐7.41 (m, 1H, Ar‐H), 7.28‐7.25 (d, 1H, Ar‐H), 7.14‐7.08 (m, 2H, Ar‐H), 6.96‐85 (m, 2H, Ar‐H);^13^C NMR (100 MHz, DMSO‐*d*_6_): *δ* 115.5, 115.7, 117.5, 120.1, 122.4, 126.3, 162.6, 168.4, 171.3, 175.5; HRMS (m/z): 303.03 Calcd. For C_14_H_10_FN_3_S_2_; found 304 (M + H)^+^; Elemental Analysis: C 65.43, H 10.32, N 3.85, S 2.44, found C 63.64, H 9.43, N 2.67, S 2.30.


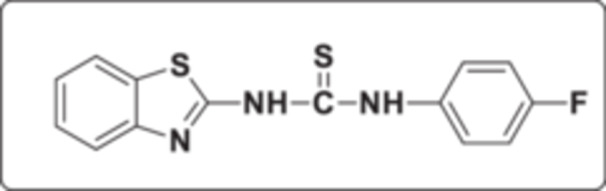


#### 2.5.8 1‐(benzo[d]thiazol‐2‐yl)‐3‐phenylthiourea (5c)

Chemical Formula: C_14_H_11_N_3_S_2_; White solid, m.p. 163‐166; Yield 92%; IR (KBr) (ν_max_/cm^‐1^): 1033, 1319, 1424, 1663, 2997, 3441); ^1^H NMR (400 MHz, DMSO‐*d*_6_, ppm): *δ* 10.51 (s, 1H, NH), 8.21 (s, 1H, NH), 7.54‐7.52 (d, 1H, Ar‐H), 7.41‐7.35 (d, 2H, Ar‐H), 7.32‐7.30 (d, 1H, Ar‐H), 7.16‐7.10 (m, 4H, Ar‐H), 7.08‐7.04 (t, 1H, Ar‐H); ^13^C NMR (100 MHz, DMSO‐*d*_6_): *δ* 116.5, 116.7, 118.5, 122.1, 123.4, 126.0, 162.5, 167.4, 170.3, 174.5; HRMS (m/z): 285.38 Calcd. For C_14_H_11_N_3_S_2_; found 286.28 (M + H)^+^; Elemental Analysis: C 58.92, H 3.89, N 14.72, S 22.47 found C 57.82, H 2.99, N 13.62, S 22.37.


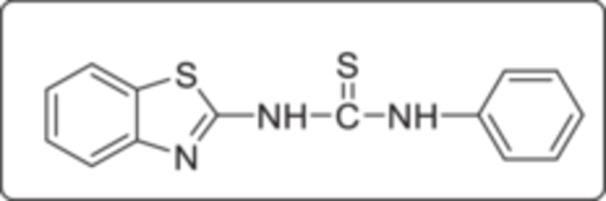


#### 2.5.9 N‐(benzo[d]thiazol‐2‐yl)‐4‐fluorobenzenesulfonamide (5 d)

Chemical Formula: C_13_H_9_FN_2_O_2_S_2_; White solid, m.p. 165‐168; Yield 92%; IR (KBr) (ν_max_/cm^‐1^): 769, 948, 1219, 1351, 1439, 1536, 1642, 2998,3447; ^1^H NMR (400 MHz, DMSO‐*d*_6_, ppm): *δ* 8.43 (s, 1H, NH), 7.82‐7.79 (m, 4H, Ar‐H), 7.55‐53 (d, 1H, Ar‐H), 7.49‐7.44 (t, 1H, Ar‐H), 7.38‐7.37 (d, 1H, Ar‐H), 7.01‐6.97 (t, 1H, Ar‐H); ^13^C NMR (100 MHz, DMSO‐*d*_6_): *δ* 114.7, 120.9, 127.9, 141.5, 162.5, 162.7, 164.7, 168.1, 176.8, 177.6, 178.4; HRMS (m/z): 308.35 Calcd. For C_13_H_9_FN_2_O_2_S_2_; found 309.28 (M + H)^+^; Elemental Analysis: C 50.64, H 2.94, N 9.09, S 20.79 found C 50.34, H 2.74, N 8.08, S 20.59.


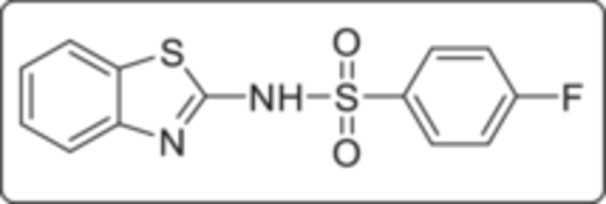


#### 2.5.10 N‐(benzo[d]thiazol‐2‐yl)‐4‐chloro‐3‐nitrobenzenesulfonamide (5e)

Chemical Formula: C_13_H_8_ClN_3_O_4_S_2_; White solid, m.p. 164‐167; Yield 92%; IR (KBr) (ν_max_/cm^‐1^): 769, 949, 1220, 1351, 1439, 1536, 1642, 2999,3446: ^1^H NMR (400 MHz, DMSO‐*d*_6_, ppm):*δ* 10.51 (s, 1H, NH), 8.21 (s, 1H, Ar‐H), 7.55‐7.53(d, 1H, Ar‐H), 7.41‐7.40 (d, 1H, Ar‐H), 7.36‐7.35 (d, 1H, Ar‐H), 7.33‐7.31 (d, 1H, Ar‐H), 7.16‐7.10 (t, 1H, Ar‐H), 7.08‐7.04 (t, 1H, Ar‐H);^13^C NMR (100 MHz, DMSO‐*d*_6_): *δ*109.7, 113.8, 123.4, 128.1, 130.7, 131.9, 144.7, 144.8, 145.3, 147.2, 149.6, 164.5, 166.7; HRMS (m/z): 368.96 Calcd. For C_13_H_8_ClN_3_O_4_S_2_; found 369.08 (M + H)^+^; Elemental Analysis: C 42.22, H 2.18, N 11.36, S 17.34 found C 42.02, H 2.08, N 11.26, S 17.14.


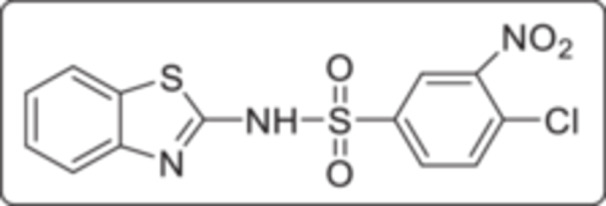


**Figure 1:**
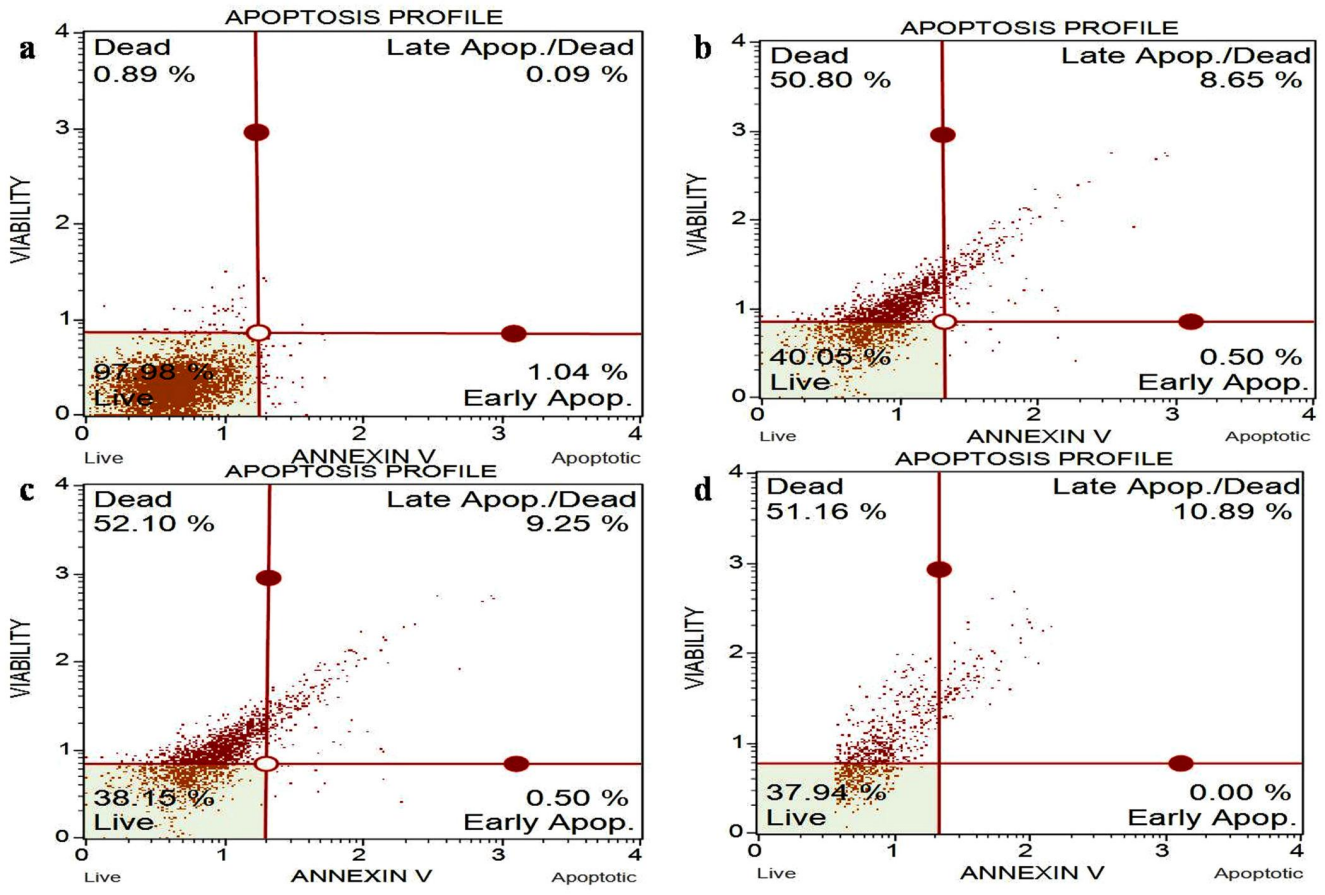
Cell apoptosis assay by cell analyzer: MDA‐MB‐231 cells (a) Control cells (untreated cells) (b) 3 d compound treated cells (c) 5b compound treated cells (d) Doxorubicin (standard drug) treated cells and all cells were stained by Annexin‐V and PI to detect apoptosis.

**Figure 2:**
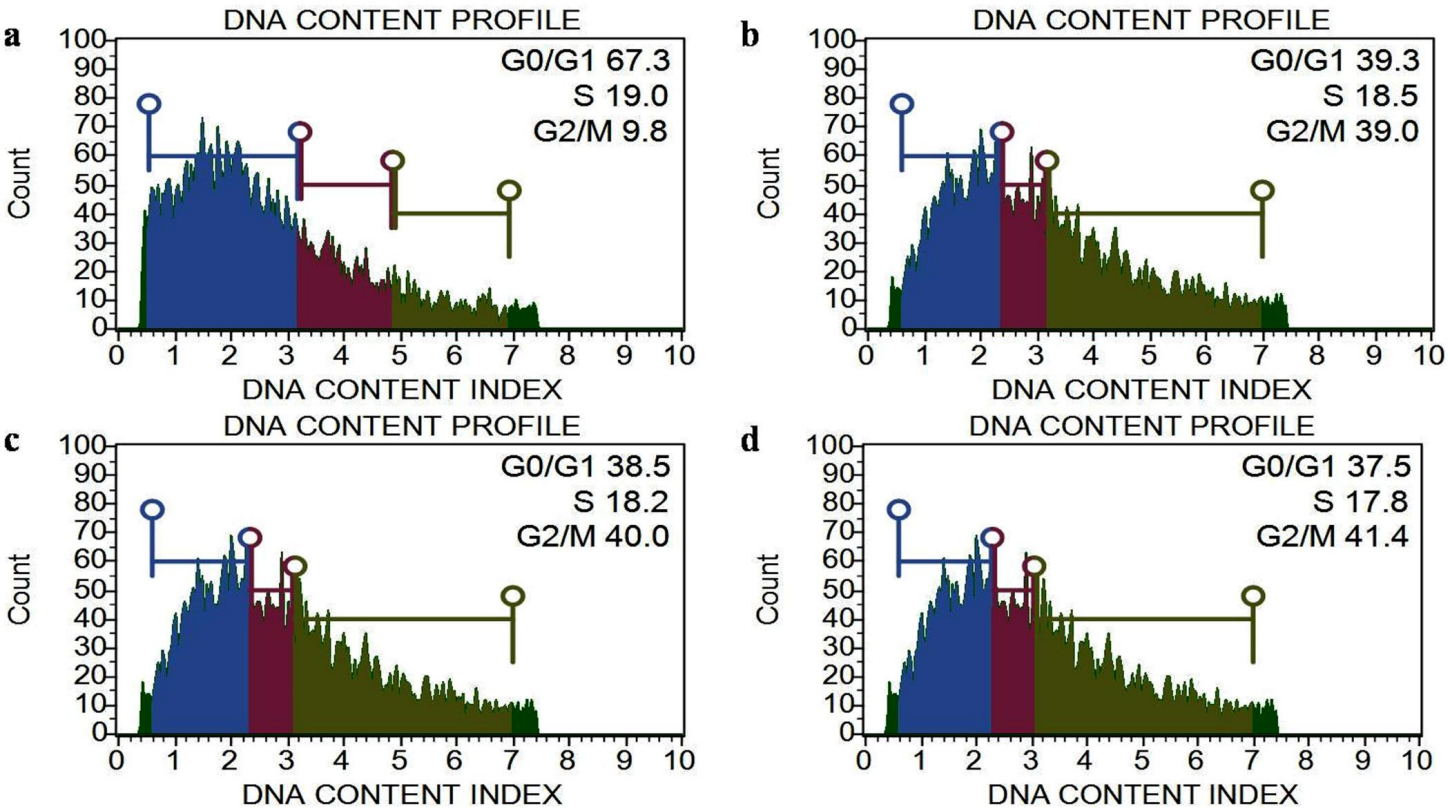
Cell Cycle progress: MDA‐MB‐231 cells (a) Control cells (untreated cells) (b) 3 d compound treated cells (c) 5b compound treated cells (d) Doxorubicin (standard drug) treated cells.

**Figure 3:**
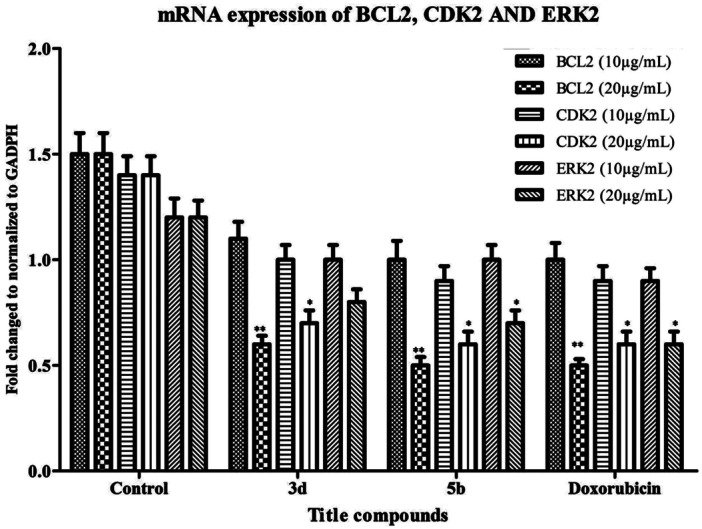
Quantitative RT‐PCR analyses of mRNA expression profile of key cancer signal marker genes at transcriptional level in control (untreated), 3 d, 5b and Doxorubicin treated MDA‐MB‐231 breast cancer cells. Data are represented as mean ± SD of triplicates, groups were compared using one‐way ANOVA test and the values are significant at *p* < 0.05.

**Figure 4:**
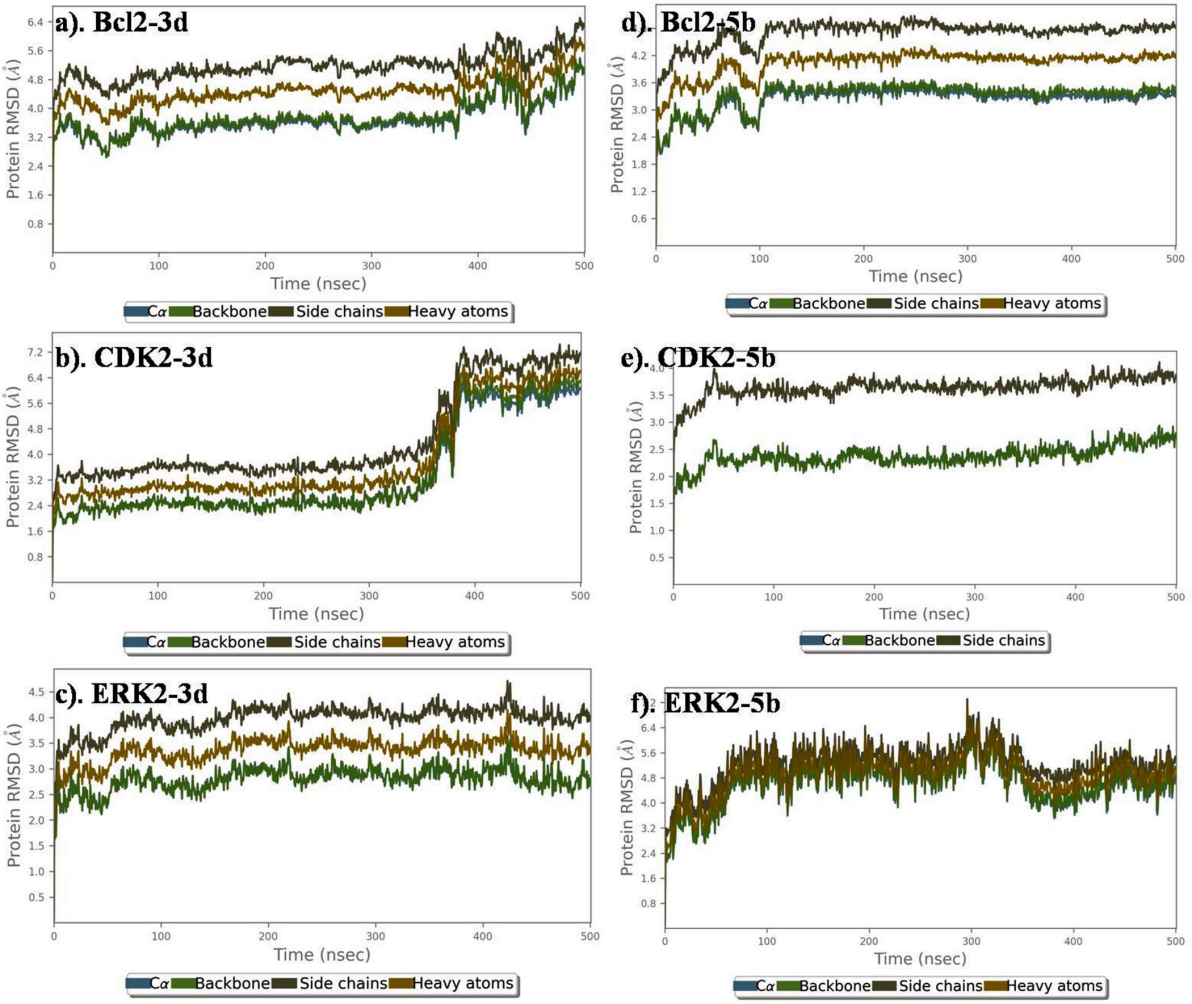
MD simulations of Root Mean Square Deviation (RMSD) results: (a) RMSD plot of Bcl2‐3d complex; (b) RMSD plot of CDK2‐3d complex; (c) RMSD plot of ERK2‐3d complex; (d) RMSD plot of Bcl2‐5b complex; (e) RMSD plot of CDK2‐5b complex and (f) RMSD plot of ERK2‐5b complex.

**Figure 5:**
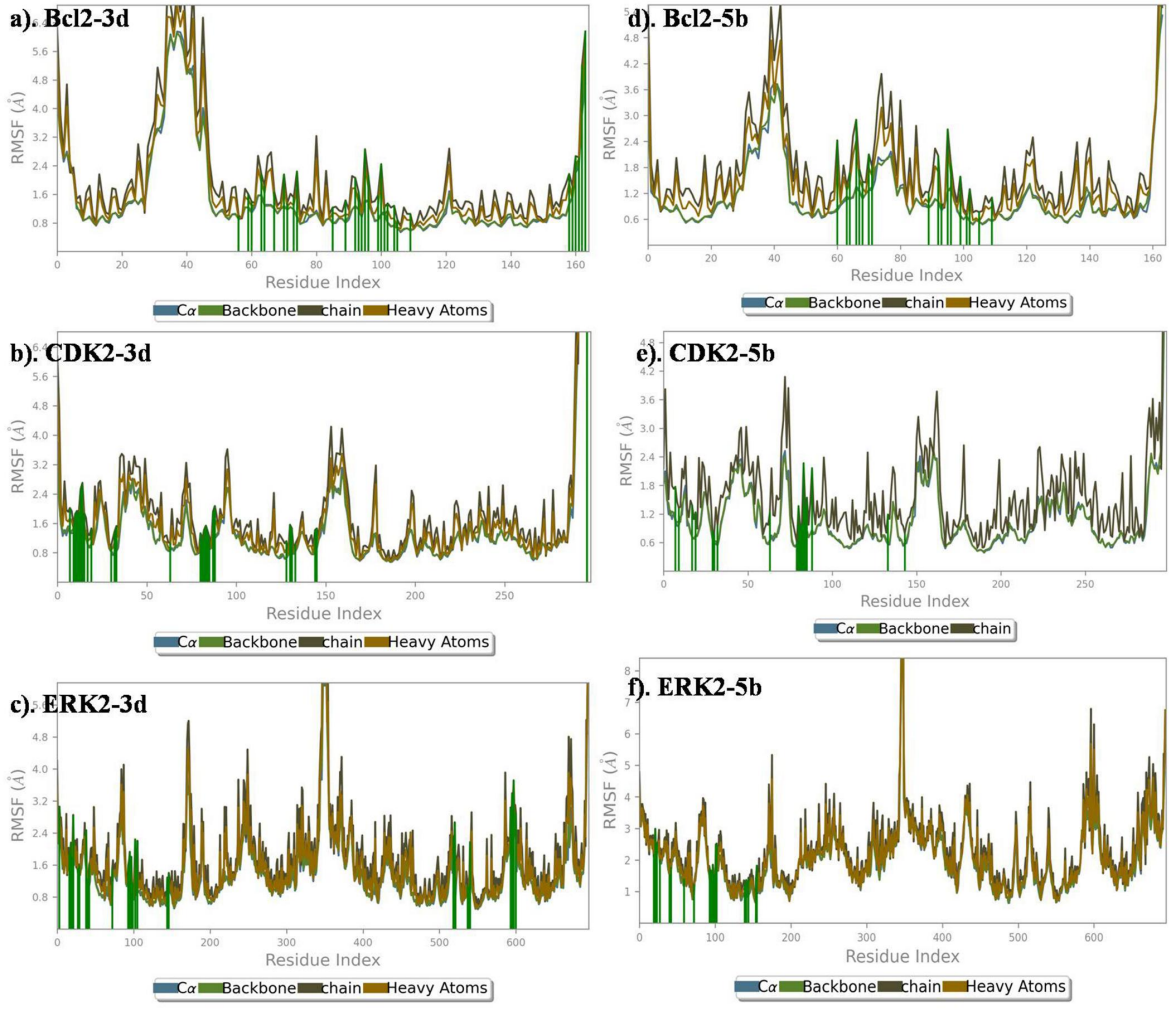
MD simulations of Protein‐Root Mean Square Fluctuation (RMSF) results: (a) RMSF plot of Bcl2‐3d complex; (b) RMSF plot of CDK2‐3d complex; (c) RMSF plot of ERK2‐3d complex; (d) RMSF plot of Bcl2‐5bcomplex; (e) RMSF plot of CDK2‐5b complex and (f) RMSF plot of ERK2‐5b complex.

The corresponding ^1^H NMR, ^13^C NMR, FT‐IR, HRMS and CHN analysis figures are provided in the **Supplementary Material** (S Figures 1–5) **to** support compound identification and ensure the reproducibility of the reported results.

### 2.6 Assessment of Antioxidant Activity

#### 2.6.1 DPPH Radical Scavenging Assay

The DPPH radical scavenging potency of newly synthesised compounds, such as **3a‐e** and **5a‐e**, was determined using the 2, 2‐diphenyl‐2‐picryl‐hydrazyl assays, which were conducted in accordance with the revised method of Venkataswamy et al [15]. A stock solution was generated by dissolving 4 milligrams of DPPH in 100 millilitres of methanol. The working standard was prepared by diluting a stock solution with methanol. Subsequently, 4 mL of the DPPH standard solution was supplemented with varying concentrations of newly synthesized compounds (10, 20, 30, and 40 μg/mL). Following this, the samples were incubated in the dark at room temperature for 20 min with 100 μL of methanol. The optical absorption at 517 nm was observed.

The absorbance of the standard ascorbic acid and the parallel control (without additives) was also evaluated using comparable methodologies. The following equation was employed to determine the DPPH radical scavenging activity.

Inhibition(%)=[(Acontrol–Atest)/Acontrol]×100.

Where;

A control = Absorbance of the control;

A test = Absorbance of the synthesized compounds.

The findings of the radical scavenging activity were expressed using the half maximal inhibitory concentration (IC_50_) in comparison to the standard. The mean and standard deviation values were determined following the completion of each measurement in triplicate.

#### 2.6.2 ABTS Radical Scavenging Assay

The antioxidant activity of the newly synthesized compounds, such as **3a‐e** and **5a‐e** were assessed using the 2,2‐azino‐bis‐3‐ethyl benzthiazoline‐6‐sulphonic acid (ABTS) radical scavenging assay, was carried out as outlined in the revised method described by Venkataswamy et al. [15] The absorbance was measured at 734 nm after 50 μL of various concentrations of newly synthesized compounds dissolved in methanol (10, 20, 30 and 40 μg/mL) were made up to 400 μL using ABTS solution and incubated at ambient temperature for 30 min. The mean and standard deviation values were computed for all measurements, which were conducted in triplicate. The extract's antioxidant capacity was determined by employing the subsequent equation, and the results were expressed as the half of the maximal inhibitory concentration (IC_50_) when compared to the standard.

$$\text{ABTS}\;\text{radical}\;\text{scavengingassay}\left(\%\right)=\left[\left(A_{\text{control}}–A_{\text{test}}\right)/A_{\text{control}}\right]\times 100$$

Where,

A_control_ = Absorbance of the control;

A_test_ = Absorbance of the newly synthesized compounds.

### 2.7 MTT Assay for Cytotoxicity

The cytotoxic potential of the newly synthesized compounds were evaluated against MDA‐MB‐231 human breast cancer cells and noncancerous 3T3‐L1 mammalian cells using the 3‐[4,5‐dimethylthiazol‐2‐yl]‐2,5‐diphenyl tetrazolium bromide (MTT) assay. Briefly, MDA‐MB‐231 and 3T3‐L1 mammalian cells were seeded into two individual 96‐well plates at a density of 1.5 × 10^4^ cells per well in 100 μL of culture medium and allowed to adhere for 24 h, achieving 70–80% confluence. Cells were then treated with increasing concentrations of the test compounds (5, 10, 20, 30, 40 and 50 μg/mL) and incubated for an additional 24 h at 37°C. Following treatment, 0.8 mg/mL of MTT solution was added to each well, and the plates were incubated for 4 h under standard conditions to allow for formazan crystal formation. After incubation, the supernatant was carefully removed, and 100 μL of dimethyl sulfoxide (DMSO) was added to solubilize the formazan product. The plates were then placed on a shaker to ensure complete dissolution, and absorbance was measured at 570 nm using a Bio‐Rad microplate reader. All treatments were performed in triplicate. Untreated cells served as the control group for baseline comparison. The percentage of cell viability relative to the control was calculated to determine compound‐induced cytotoxicity [16].

### 2.8 Cell Apoptosis Measurement

MDA‐MB‐231 cells were seeded into individual 6‐well plates at a density of 1.5 × 10^4^ cells per well and incubated for 24 h to reach approximately 70–80% confluence. Cells were then treated with the newly synthesized compound at a final concentration of 20 μg/mL and incubated for an additional 24 h at 37°C. Following treatment; cells were washed with TE buffer and incubated with 5 μL of Annexin V reagent and propidium iodide (PI) in the dark for 30 min at room temperature. Apoptotic cells were quantified using a flow‐based cell analyzer, and the results were expressed as the percentage of fluorescence‐positive cells, based on the manufacturer's instructions [17].

### 2.9 Cell Cycle Analysis

MDA‐MB‐231 cells were seeded into 6‐well plates at a density of 1.5 × 10^4^ cells per well and allowed to adhere for 24 h, reaching approximately 70–80% confluence. Following incubation, cells were washed three times with phosphate‐buffered saline (PBS) and treated with the newly synthesized compound at a concentration of 20 μg/mL. Treated cells were then incubated at 37°C for an additional 24 h. Post‐treatment, cells were harvested using trypsin‐EDTA, and subsequently fixed in 70% ethanol (30 mL) at 4°C overnight. Fixed cells were pelleted by centrifugation at 1500 rpm for 5 min, then resuspended in 200 μL of PBS containing 0.1% Triton X‐100 and RNase A (final concentration: 100 μg/mL), followed by incubation at 37°C for 2 h. After a second centrifugation at 1500 rpm for 5 min at 4°C, the cell pellets were stained with propidium iodide (PI) at a final concentration of 30 μg/mL and incubated in the dark at room temperature for 30 min. Cell cycle distribution was analyzed using a Muse™ Cell Analyzer (Merck Millipore, USA), and data were processed following standard protocols [18].

### 2.10 Expression of Signal Transduction Marker Genes by qRT‐PCR Analysis

Quantitative real‐time polymerase chain reaction (qRT‐PCR) analysis was carried out to evaluate the mRNA expression levels of key signal‐transduction‐related genes, namely BCL2, CDK2, and ERK2, in untreated (control) and compound‐treated MDA‐MB‐231 cells. The cells were exposed to the test compounds at concentrations of 10 and 20 μg/mL, while doxorubicin served as a reference control. Total RNA was isolated using TRI Reagent® (Sigma‐Aldrich, USA) following the manufacturer's recommended protocol. The concentration and purity of RNA were determined spectrophotometrically by measuring the A260/A280 ratio, and RNA integrity was confirmed by agarose gel electrophoresis. Subsequently, 1 μg of total RNA was reverse‐transcribed into complementary DNA (cDNA) using a High‐Capacity cDNA Reverse Transcription Kit (Applied Biosystems, Foster City, CA, USA). The qRT‐PCR amplification was performed using a Bio‐Rad CFX96 Real‐Time PCR Detection System (Bio‐Rad, USA) with SYBR™ Green Master Mix (Applied Biosystems, USA). Each reaction mixture contained approximately 20 ng of cDNA template and gene‐specific primers. The thermal cycling conditions consisted of an initial denaturation at 95°C for 30 s, followed by 40 cycles of denaturation at 95°C for 5 s and annealing/extension at 60°C for 30 s. To ensure reliability and reproducibility, three independent biological replicates were included for each experimental condition, and each biological sample was analyzed intechnical triplicates. Primer specificity was validated by melt‐curve analysis, which produced a single sharp peak, confirming the absence of nonspecific amplification or primer‐dimer formation. The primers generated amplicons of approximately 100–200 bp, and amplification efficiency was assessed using a standard dilution curve. Only primer sets exhibiting efficiencies between 90% and 110% were used for subsequent analyses. Gene expression levels were normalized using GAPDH as the internal reference gene, and its expression stability across experimental treatments was verified prior to normalization. Relative gene expression was calculated using the comparative threshold cycle (ΔΔCt) method, and fold changes in expression were determined using the 2^n^‐ΔΔCt algorithm. Gene‐specific primers used for amplification are listed below

Bcl2 is:

5′‐ATTGGGAAGTTTCAAATCAGC‐3’ (Forward),

5′‐CAGTCTACTTCCTCTGTGATGTTG‐3′ (Reverse),

CDK2 is

5′‐ATGGATGCCTCTGCTCTCACTG‐3′ (Forward),

5′‐CCCGATGAGAATGGCAGAAAGC‐3′ (Reverse),

ERK2 is

5′‐ACACCAACCTCTCGTACATCGG‐3′ (Forward),

5′‐TGGCAGTAGGTCTGGTGCTCAA‐3′ (Reverse),

GAPDH is:

5′‐ACCACAGTCCATGCCATCAC‐3′ (Forward),

5′‐TCCACCACCCTGTTGCTG TA‐3′ (Reverse) and transcription levels of everygene were normalised to the level of GAPDHgene.

### 2.11 Glide Docking Procedure and Prime MM‐GBSA

The three‐dimensional structures of CDK2 (PDB ID: 1AQ1), BCL2 (PDB ID: 1YSW), and ERK2 (PDB ID: 4QP2) were retrieved from the Protein Data Bank. In cases where crystal structures were incomplete or unavailable, homology modelling was performed using a comparative modelling approach to construct the full tertiary structure [19]. Rigid Receptor Docking (RRD) was utilized to evaluate the binding interactions and docking scores between the synthesized ligands and the target proteins CDK2, BCL2, and ERK2. Receptor grids were generated around the active sites of each protein using the Glide v6.3 module (Schrödinger LLC), with default parameters including a van der Waals radius scaling factor of 1.00 and a partial charge cutoff of 0.25. Ligands and receptor structures were prepared and imported into the Maestro v9.8 interface. Docking was initially performed using Glide Standard Precision (SP) mode to generate multiple ligand poses. These were subsequently refined using the QSite module for QM/MM (quantum mechanics/molecular mechanics) optimization. Partial atomic charges were computed for each pose to improve accuracy in energy estimations. For high‐precision docking, the most energetically favourable ligand conformations were re‐docked using Glide Extra Precision (XP) mode, incorporating the optimized charge sets. Binding free energies (ΔG) of the ligand–protein complexes were further estimated using the Prime MM‐GBSA method, which offers improved predictive accuracy over standard docking scores [17, 20]. The final analysis included docking score assessment, interaction mapping, and binding affinity prediction of ligand series **3(a–e)** and **5(a–e)** with CDK2, BCL2, and ERK2 targets, providing insight into their potential molecular mechanisms of action.

### 2.12 Molecular Dynamics Simulations

To evaluate the dynamic behavior and structural stability of the top‐ranked protein–ligand complexes, molecular dynamics (MD) simulations were performed using the Desmond v5.7 simulation package (Schrödinger LLC). Simulations were carried out for a total duration of 150 ns on selected complexes of CDK2, BCL2, and ERK2 with compounds **3 d** and **5b**. Each protein–ligand complex was solvated in an orthorhombic box with explicit water molecules using the TIP3P water model, and system neutrality was achieved by adding appropriate counter ions. Simulations were conducted under physiological conditions, with the temperature maintained at 300 K and pressure at 1 atm using the NPT ensemble. The structural stability and conformational changes of the complexes were assessed by monitoring the root‐mean‐square deviation (RMSD) and root‐mean‐square fluctuations (RMSF) of the protein backbone and ligand atoms throughout the simulation trajectory. Additionally, the radius of gyration (rGyr) was analyzed to evaluate protein compactness during the simulation period. Protein–ligand interactions, including hydrogen bonds, hydrophobic contacts, and water bridges, were tracked over time to understand the binding stability and molecular interaction profile. The performance of the ligand‐bound complexes was compared to their respective co‐crystal structures under identical simulation conditions to validate binding efficiency [19].

### 2.13 Statistical Analysis

All experiments were performed in triplicate, and results are expressed as mean ± standard deviation (SD). Statistical analyses were conducted using GraphPad Prism version 8.0 (GraphPad Software, USA) and Microsoft Excel. One‐way analysis of variance (ANOVA) was employed to evaluate differences among multiple groups. A p‐value of less than 0.05 was considered statistically significant. The IC_50_ values were calculated by nonlinear regression analysis using a sigmoidal dose‐response (four‐parameter logistic) model in GraphPad Prism version 8.0.

## 3 Results

### 3.1 Chemistry

#### 3.1.1 Synthesis of 4‐(4‐amino‐3‐fluorophenoxy)‐N‐methylpicolinamide and 2‐Aminobenzothiazole Derivatives of 3a‐e and 5a‐e

A new series of 4‐(4‐amino‐3‐fluorophenoxy)‐N‐methylpicolinamide **(1)** (0.001 mol) in THF (10 mL) and triethylamine (0.001 mol), as a base with various phenyl isocyanates, isothiocyanates, and sulfonamides **(2a‐e)** (0.001 mol) was added at 10°C. The reaction was slowly raised to temperature 60°C while stirring the mixture for 4 h. Every half an hour, TLC was used to evaluate the reaction progress using a mobile phase of n‐hexane: ethylacetate (3:1). After completion of the reaction, to get solid products **(3a‐e).** All The products were shown in Scheme 1. All the title products were subjected to column chromatography with a ratio of 3:1 of n‐hexane and ethylacetate. Another new series of title compounds **5(a‐e)** were synthesized using the same pathway, which included reacting with **2‐Aminobenzothiazole** (**4)** with various phenyl isocyanates, isothiocyanates and sulfonamides **(2a‐e)** shown in Scheme 1. After the completion of the reaction, the products were purified by using n‐hexane and ethyl acetate to get pure compounds with good yields. All the title compounds were confirmed and characterized by using FT‐IR, ^1^H NMR, ^13^C NMR, LCMS and CHN analysis studies (S Figures 1–5).

The data on the physical characteristics of the newly synthesised compounds including 4‐(4‐amino‐3‐fluorophenoxy)‐N‐methylpicolinamide and 2‐Aminobenzothiazole derivatives are reported in **Supplementary data**(S Table 1).

### 3.2 Assessment of Antioxidant Activity

#### 3.2.1 DPPH Free Radical Scavenging Assay

The antioxidant potential of the synthesized fluorophenoxy‐picolinamide and aminobenzothiazole derivatives was evaluated using the DPPH free radical scavenging assay. Among all tested compounds, derivatives 3 d and 5b exhibited the highest radical scavenging activity at a concentration of 20 μg/mL, with inhibition values of 45.58 ± 2.62% and 49.86 ± 2.84%, respectively. The IC_50_ values of compounds 3 d and 5b were 21.45 μg/mL and 20.05 μg/mL, which are comparable to the standard antioxidant ascorbic acid (IC_50_ = 19.18 μg/mL). The lower IC_50_ values indicate a stronger free radical scavenging potential of these derivatives (Table 1).

**Table 1:** The *In Vitro* free radical scavenging activity of the 3a‐e and 5a‐e title compounds by DPPH method.

Compound	10 µg/mL	20 µg/mL	30 µg/mL	40 µg/mL	IC_50_ µg/mL
3a	19.44 ± 1.18	31.47 ± 1.63	43.11 ± 2.34	54.20 ± 2.77	36.90
3b	18.26 ± 1.05	29.63 ± 1.61	41.32 ± 1.97	52.04 ± 2.21	38.43
3c	25.71 ± 1.46	36.45 ± 2.21	47.68 ± 2.49	58.52 ± 2.60	31.45
3 d	33.18 ± 2.14	45.58 ± 2.62	57.23 ± 2.83	69.45 ± 3.41	21.45
3e	26.09 ± 1.22	37.13 ± 2.76	48.84 ± 2.62	56.42 ± 2.92	30.71
5a	24.47 ± 1.34	35.58 ± 1.92	45.61 ± 2.51	66.77 ± 3.16	32.88
5b	37.23 ± 2.21	49.86 ± 2.84	62.09 ± 3.36	75.19 ± 4.11	20.05
5c	19.23 ± 1.26	30.81 ± 2.66	42.62 ± 2.29	53.51 ± 2.70	37.37
5 d	19.69 ± 1.29	31.06 ± 2.52	42.96 ± 3.52	53.91 ± 2.84	37.09
5e	26.69 ± 1.28	37.68 ± 2.71	49.06 ± 2.71	67.11 ± 3.22	30.57
Ascorbic acid	39.07 ± 2.40	52.13 ± 3.12	63.75 ± 3.46	77.66 ± 4.31	19.18

*Note:* Data represents Mean ± S.D of triplicates.

#### 3.2.2 2, 2‐azino‐bis‐3‐ethyl benzthiazoline‐6‐sulphonic Acid (ABTS) Free Radical Scavenging Activity

The 2, 2‐azino‐bis‐3‐ethyl benzthiazoline‐6‐sulphonic acid (ABTS) free radical scavenging potential of the synthesized fluorophenoxy‐picolinamide and aminobenzothiazole derivatives was evaluated to assess their antioxidant efficacy. Among the tested compounds, 3 d and 5b demonstrated the most pronounced scavenging activity at a concentration of 20 μg/mL, with inhibition percentages of 44.96 ± 2.51% and 45.48 ± 2.35%, respectively. The IC_50_ values of compounds 3 d and 5b were determined to be 22.24 μg/mL and 21.98 μg/mL, which are comparable to that of the standard antioxidant ascorbic acid (IC_50_ = 20.04 μg/mL). The relatively low IC_50_ values indicate that these derivatives possess significant nitric oxide radical scavenging potential (Table 2).

**Table 2:** The *In Vitro* free radical scavenging activity of the 3a‐e and 5a‐e title compounds by ABTS method.

Compound	10 µg/mL	20 µg/mL	30 µg/mL	40 µg/mL	IC_50_ µg/mL
3a	17.25 ± 1.03	26.36 ± 1.21	35.17 ± 2.02	43.45 ± 2.77	46.02
3b	16.23 ± 0.91	24.31 ± 1.22	31.26 ± 1.24	40.35 ± 2.01	49.56
3c	20.11 ± 1.04	29.41 ± 1.42	38.61 ± 2.11	47.23 ± 2.97	42.34
3 d	32.63 ± 2.09	44.96 ± 2.51	55.49 ± 2.66	66.19 ± 3.27	22.24
3e	21.09 ± 1.29	30.56 ± 2.38	39.74 ± 2.20	48.29 ± 2.99	41.41
5a	22.32 ± 1.10	32.74 ± 1.88	42.52 ± 2.39	63.23 ± 3.06	35.277
5b	33.26 ± 2.04	45.48 ± 2.35	57.89 ± 3.11	68.61 ± 3.77	21.98
5c	18.01 ± 1.12	28.22 ± 2.42	40.56 ± 2.13	50.66 ± 2.72	39.47
5 d	18.27 ± 1.21	30.24 ± 2.47	40.82 ± 3.30	50.46 ± 2.66	39.63
5e	23.56 ± 1.11	34.19 ± 2.43	46.45 ± 2.19	64.37 ± 3.02	32.29
Ascorbic acid	37.27 ± 2.22	49.88 ± 3.09	59.45 ± 3.29	71.33 ± 4.20	20.04

*Note:* Data represents Mean ± S.D of triplicates.

### 3.3 Cytotoxicity by MTT Assay

**Figure 6:**
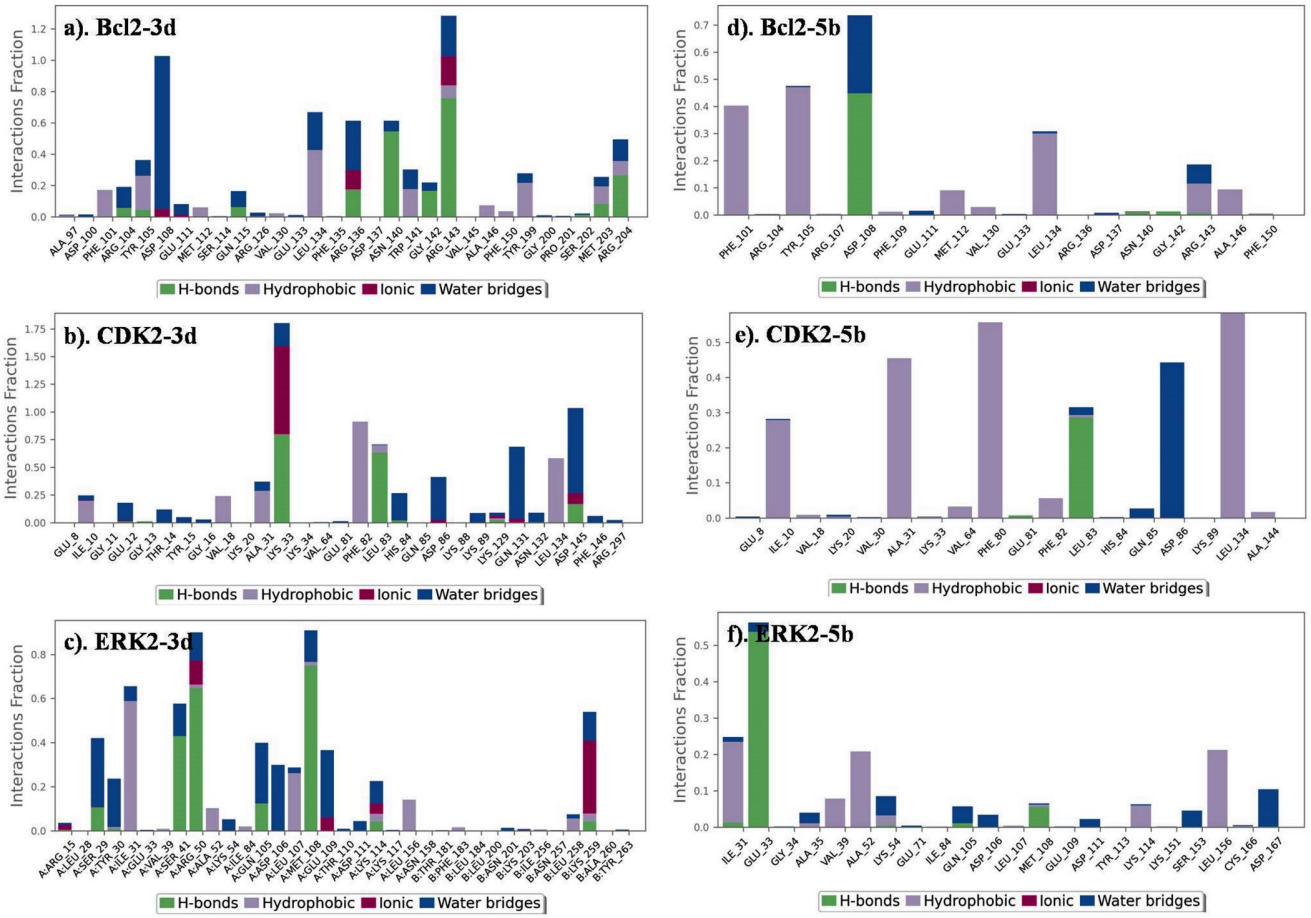
MD simulations of active site residues involved in the bonded and non‐bonded contacts (a) Bcl2‐3d complex; (b) CDK2‐3d complex; (c) ERK2‐3d complex; (d) Bcl2‐5b complex; (e) CDK2‐5b complex and (f) ERK2‐5b complex.

The cytotoxic potential of the synthesized compounds **(3a‐e** and **5a‐e**) was evaluated against MDA‐MB‐231 human breast cancer cells and noncancerous 3T3‐L1 mammalian cells using the MTT assay at varying concentrations (5, 10, 20, 30, 40, and 50 μg/mL) for a 24‐h exposure period. The corresponding results are summarized in Table 3. All tested derivatives exhibited moderate to potent cytotoxic effects against MDA‐MB‐231 cells. Among them, compounds 3 d and 5b displayed the most significant activity, with IC_50_ values of 22.13 μg/mL and 20.11 μg/mL, respectively, which are comparable to the standard reference drug doxorubicin (IC_50_ = 19.02 μg/mL). The lower IC_50_ values observed for these compounds indicate stronger antiproliferative efficacy against breast cancer cells. In contrast, treatment with compounds **3a–e and 5a–e** showed no evident cytotoxicity in noncancerous 3T3‐L1 mammalian cells (S Table 2), confirming the selectivity of the test compounds and the results were summarized in Supplementary Data, S Figure 6E–H.

**Table 3:** Effect of synthesized novel 3a‐e and 5a‐e title compounds on percentage of inhibition of MDA‐MB‐231 cell viability by MTT assay.

Compounds	% of inhibition of MDA MB‐231cells						
	5 µg/mL	10 µg/mL	20 µg/mL	30 µg/mL	40 µg/mL	50 µg/mL	IC_50_ µg/mL
3a	9.25 ± 1.1	25.39 ± 2.25	40.18 ± 2.72	53.41 ± 2.88	69.18 ± 3.37	82.16 ± 4.85	28.08
3b	8.86 ± 1.1	23.59 ± 2.11	37.06 ± 2.26	49.22 ± 2.62	67.56 ± 3.21	80.19 ± 4.21	30.47
3c	9.33 ± 1.2	27.55 ± 2. 31	42.95 ± 2.82	55.18 ± 3.10	69.22 ± 3.65	83.16 ± 5.03	27.18
3 d	11.56 ± 1.5	32.06 ± 2.52	45.18 ± 3.06	58.41 ± 3.23	74.66 ± 3.72	88.62 ± 5.66	22.13
3e	9.48 ± 1.3	28.18 ± 2.36	43.50 ± 2.21	55.86 ± 3.32	70.44 ± 3.62	84.18 ± 5. 31	26.85
5a	9.21 ± 1.1	25.55 ± 2.16	38.29 ± 2.37	52.80 ± 2.73	68.46 ± 3.42	81.42 ± 4.49	28.40
5b	12.02 ± 1.7	34.65 ± 2.74	49.72 ± 3.45	62.04 ± 3.07	76.81 ± 3.98	92.11 ± 6.35	20.11
5c	7.66 ± 0.9	21.72 ± 2.02	36.46 ± 2.11	48.15 ± 2.31	65.58 ± 3.08	77.06 ± 3.88	31.15
5 d	9.29 ± 1.2	26.18 ± 2.29	43.42 ± 2.95	56.01 ± 3.32	70.19 ± 3.23	83.97 ± 4.86	26.78
5e	9.02 ± 1.1	24.11 ± 2.35	39.11 ± 2.64	52.44 ± 2.91	68.32 ± 3.40	81.14 ± 4.33	28.60
Doxorubicin	12.06 ± 1.8	35.22 ± 2.83	52.56 ± 3.71	64.15 ± 3.25	78.08 ± 4.26	96.19 ± 7.22	19.02

### 3.4 Cell Viability by Microscopic Assay

Fluorescence microscopy further supported the cytotoxic effects observed in the MTT assay. Treatment of MDA‐MB‐231 breast cancer cells and 3T3‐L1 noncancerous cells with synthesized compounds (3a‐e and 5a‐e) resulted in a marked reduction in cell viability, indicating moderate to strong cytotoxic activity. Notably, compounds 3 d and 5b exhibited a significant, concentration‐dependent decrease in viability in MDA‐MB‐231 cells, while showing no observable effect on 3T3‐L1 cells. Fluorescence images of treated and untreated cells (**Supplementary Data,** Figure S 6A–D) demonstrated that exposure to compounds 3 d and 5b (10–40 μg/mL) substantially reduced viable cancer cell populations, comparable to the standard drug doxorubicin.

### 3.5 Apoptosis and Cell Death Analysis

The apoptotic profile and cell death were assessed using a cell analyzer with Annexin V and propidium iodide (PI) staining. MDA‐MB‐231 cells treated for 24 h with 10–40 μg/mL concentrations of the newly synthesized 3a‐e and 5a‐e compounds exhibited significant (*p* < 0.05) changes in live, early apoptotic, late apoptotic, and necrotic cell populations compared to control cells. In untreated control cells, the percentage of viable cells was 97.98% and late apoptosis was 0.09%. In contrast, treatment with compounds 3 d and 5b (20 μg/mL) reduced cell viability to 40.06% and 38.15%, late apoptosis increased to 8.75% and 9.25 respectively, the values comparable to those observed in doxorubicin‐treated cells' late apoptosis (10.89%), as illustrated in Figure 1a‐d. These findings indicate that compounds 3 d and 5b significantly diminished the proportion of viable cells while inducing late apoptosis at levels similar to the standard chemotherapeutic agent doxorubicin.

### 3.6 Cell Cycle Analysis

The cell‐cycle progression of MDA‐MB‐231 breast cancer cells was analyzed using a flow cytometry‐based cell analyzer following treatment with the synthesized compounds **3a‐e** and **5a‐e**. Notably, cells exposed to compounds 3 d and 5b at a concentration of 20 μg/mL exhibited a marked alteration in DNA content distribution compared to the untreated control group. Specifically, the proportion of cells in the G0/G1 phase decreased from 67.3% to 39.3% and 38.5%, while those in the S phase were reduced from 19.0% to 13.4% and 13.1%, respectively. Conversely, a significant accumulation of cells was observed in the G2/M phase, increasing from 9.8% to 39.0% and 40.0%, respectively (Figure 2a‐d). These findings indicate that both 3 d and 5b induce a pronounced G2/M phase arrest, closely resembling the cell‐cycle modulation observed in doxorubicin‐treated MDA‐MB‐231 cells.

### 3.7 Effect of Novel Synthesized Compounds on Expression of BCL2, CDK2 and ERK2 Genes by qRT‐PCR Analysis

The effects of the test compounds on the expression of key signal transduction–related genes were evaluated in MDA‐MB‐231 cells by qRT‐PCR (Figure 3). The relative mRNA levels of BCL2, CDK2, and ERK2 were quantified and normalised to GAPDH using the ΔΔCt method. Untreated control cells exhibited higher basal expression of all three genes. Upon treatment with the test compound (3 d), a clear down‐regulation of BCL2, CDK2, and ERK2 transcripts was observed, with a more pronounced reduction at 20 μg/mL compared with 10 μg/mL, indicating a concentration‐dependent effect. Specifically, BCL2 expression was markedly suppressed, suggesting inhibition of anti‐apoptotic signalling. Similarly, CDK2 mRNA levels were significantly decreased, reflecting potential interference with cell‐cycle progression. The expression of ERK2, a key component of the MAPK pathway, was also reduced following treatment, indicating attenuation of proliferative signalling pathways. Cells treated with the reference drug doxorubicin showed comparable or stronger down‐regulation of these genes, validating the responsiveness of the assay. Overall, the qRT‐PCR results demonstrate that compound 3 d effectively modulates the transcription of critical genes involved in cell survival, cell‐cycle regulation, and signal transduction in MDA‐MB‐231 cells, supporting its potential anticancer activity.

### 3.8 RRD Docking Interaction Analysis

The molecular docking studies of the novel fluorophenoxy‐picolinamide and aminobenzothiazole derivatives with the Bcl‐2 protein (PDB ID: 1YSW) demonstrated favourable binding affinities, as indicated by the calculated binding free energy (ΔGbind), extra‐precision glide (XPG) scores, and Prime MM‐GBSA results (Table 4). These computational outcomes revealed significant ligand–receptor interactions supporting strong binding potential. Among the tested compounds, compound 3 d exhibited the most stable interaction with Bcl‐2, displaying an MM‐GBSA energy of −64.66 kcal/mol, an XPG score of −5.278, and a Glide Emodel value of −59.41. The molecular interaction profile showed one strong hydrogen bond with Glu133, two π–π stacking interactions involving the aromatic rings of Phe101 and Tyr105, and multiple van der Waals contacts with residues Asp100, Phe101, Arg104, Tyr105, Asp108, Phe109, Met112, Val130, Val131, Glu133, Leu134, Gly142, Arg143, Val145, Asp146, Glh149, and Phe150 (Table 4).

**Table 4:** Docking scores, binding free energies, and molecular interaction parameters of synthesized 3a–e and 5a–e compounds with BCL2 (PDB ID: 1YSW).

Compound	QPLD Score			Interactions		Amino acids
	MM‐GBSA (ΔG_bind_)	XPG/Docking Score	Glide Emodel	H‐bonds	Pi‐pi‐cation	
3a	−49.08	−4.225	−50.473	—	3‐Bonds (Aromatic ring‐Phe101, Aromatic ring‐Phe101 Aromatic ring‐Tyr105)	Ala97, Asp100, Phe101, Arg104, Tyr105, Phe109, Met112, Val130, Glu133, Leu134, Gly142, Arg143, Val145, Asp146, Glh149, Phe150, Val153
3b	−51.87	−5.037	−53.565	2‐Bonds (N‐Glu133, O‐Arg143)	1‐Bond (Aromatic ring‐Tyr105)	Ala97, Phe101, Tyr105, Phe109, Met112, Val130, Glu133, Leu134, Gly142, Arg143, Val145, Asp146, Glh149, Phe150
3c	−54.92	−4.524	−51.972	1‐Bond (HN‐Gly142)	1‐Bond (Aromatic ring‐Tyr105)	Phe101, Tyr105, Phe109, Met112, Val130, Glu133, Leu134, Trp141, Gly142, Arg143, Val145, Asp146, Glh149, Phe150, Val153
3 d	−64.66	−5.278	−59.41	1‐Bond (N‐Glu133)	2‐Bonds (Aromatic ring‐Phe101, Aromatic ring‐Tyr105)	Asp100, Phe101, Arg104, Tyr105, Asp108, Phe109, Met112, Val130, Val131, Glu133, Leu134, Gly142, Arg143, Val145, Asp146, Glh149, Phe150
3e	−46.29	−4.475	−51.43	2‐Bonds (HN‐Tyr105, HN‐Glu133)	—	Phe101, Tyr105, Asp108, Phe109, Met112, Val130, Val131, Glu133, Leu134, Gly142, Arg143, Val145, Asp146, Asp147, Glh149, Phe150
5a	−37.31	−4.649	−47.885	—	1‐Bond (Aromatic ring‐Tyr105)	Phe101, Tyr105, Asp108, Phe109, Met112, Val130, Glu133, Leu134, Arg143, Asp146, Glh149, Phe150, Val153
5b	−44.73	−5.612	−56.858	—	1‐Bond (Aromatic ring‐Tyr105)	Phe101, Tyr105, Phe109, Met112, Val130, Glu133, Leu134, Gly142, Arg143, Val145, Asp146, Glh149, Phe150, Val153
5c	−43.35	−5.063	−42.065	—	1‐Bond (Aromatic ring‐Tyr105)	Phe101, Tyr105, Phe109, Met112, Val130, Glu133, Leu134, Gly142, Arg143, Val145, Asp146, Glh149, Phe150, Val153
5 d	−37.68	−4.283	−35.036	—	1‐Bond (Aromatic ring‐Phe101)	Phe101, Tyr105, Asp108, Phe109, Met112, Val130, Glu133, Leu134, Gly142, Arg143, Asp146, Glh149
5e	−38.13	−3.741	−47.775	—	1‐Bond (Aromatic ring‐Arg143)	Phe101, Tyr105, Asp108, Phe109, Met112, Val130, Glu133, Leu134, Gly142, Arg143, Asp146, Glh149, Phe150

Similarly, compound 5b demonstrated a strong binding orientation with an MM‐GBSA energy of −44.73 kcal/mol, an XPG score of −5.612, and a Glide Emodel value of −56.858. The interaction network revealed a prominent π–π stacking interaction between its aromatic ring and Tyr105, supported by van der Waals interactions with Phe101, Tyr105, Phe109, Met112, Val130, Glu133, Leu134, Gly142, Arg143, Val145, Asp146, Glh149, Phe150, and Val153 (Table 4). These results suggest that the designed derivatives, particularly 3 d and 5b, possess a strong affinity toward Bcl‐2, indicating their potential as promising anti‐apoptotic modulators.

The molecular docking studies of the novel fluorophenoxy‐picolinamide and aminobenzothiazole derivatives with the CDK2 and ERK2 proteins (PDB ID: 1AQ1 and 4QP2) demonstrated favourable binding affinities, as indicated by the calculated binding free energy (ΔGbind), extra‐precision glide (XPG) scores, and Prime MM‐GBSA results (Tables 5 and 6). These computational outcomes revealed significant ligand–receptor interactions supporting strong binding potential. Among the tested compounds, compound 3 d exhibited the most stable interaction with CDK2 and ERK2, displaying an MM‐GBSA energy of −51.74 and −35.28 kcal/mol, an XPG score of −7.107 and −7.764, and a Glide Emodel value of −72.329 and −65.067, respectively. The molecular interaction profile showed two strong hydrogen bond with O‐Lys33 and O‐Asp145 (CDK2) and one bond hydrogen bond with O‐Met108. Where one π–π stacking interactions involving the Aromatic ring‐IMD402 (ERK2), and multiple van der Waals contacts with residues Ile10, Gly11, Glu12, Gly13, Val18, Ala31, Lys33, Val64, Phe80, Glu81, Phe82, Leu83, His84, Gln85, Asp86, Gln131, Asn132, Leu134, Ala144, Asp145, Leu148 (CDK2) and Ile31, Gly32, Val39, Ser41, Arg50, Ala52, Lys54, Ile84, Asp106, Leu107, Met108, Glu109, Phe183, IMD402 (ERK2).

**Table 5:** Docking scores, binding free energies, and molecular interaction parameters of synthesized 3a–e and 5a–e compounds with CDK2 (PDB ID: 1AQ1).

Compound	QPLD Score			Interactions		Amino acids
	MM‐GBSA (ΔG_bind_)	XPG/Docking Score	Glide Emodel	H‐bonds	Pi‐pi‐cation	
3a	−47.50	−5.341	−51.529	4‐bonds (O‐Leu83, O‐Asp86, N‐Asp86, HN‐Gln131)	—	Ile10, Gly11, Glu12, Val18, Ala31, Val64, Phe80, Glu81, Phe82, Leu83, His84, Gln85, Asp86, Lys89, Gln131, Asn132, Leu134
3b	−44.80	−6.076	−55.086	1‐Bond (O‐Lys33)	1‐Bond (Aromatic ring‐Phe80)	Ile10, Gly11, Glu12, Gly13, Thr14, Val18, Ala31, Lys33, Val64, Phe80, Glu81, Phe82, Leu83, Gln85, Asp86, Lys89, Gln131, Asn132, Leu134, Ala144, Asp145
3c	−37.71	−5.729	−52.534	1‐Bond (O‐Lys33)	1‐Bond (Aromatic ring‐Phe80)	Ile10, Gly11, Glu12, Gly13, Thr14, Val18, Ala31, Lys33, Val64, Phe80, Glu81, Phe82, Leu83, His84, Gln85, Asp86, Gln131, Leu134, Ala144, Asp145, Phe146, Leu148
3 d	−51.74	−7.107	−72.329	2‐Bonds (O‐Lys33, O‐Asp145)	—	Ile10, Gly11, Glu12, Gly13, Val18, Ala31, Lys33, Val64, Phe80, Glu81, Phe82, Leu83, His84, Gln85, Asp86, Gln131, Asn132, Leu134, Ala144, Asp145, Leu148
3e	−41.70	−6.850	−58.591	2‐Bonds (O‐Lys33, O‐Leu83)	—	Ile10, Gly11, Glu12, Gly13, Thr14, Val18, Ala31, Lys33, Val64, Phe80, Glu81, Phe82, Leu83, His84, Gln85, Asp86, Gln131, Asn132, Leu134, Ala144, Asp145
5a	−46.98	−6.849	−53.912	—	1‐Bond (Aromatic ring‐Phe80)	Ile10, Gly11, Val18, Ala31, Lys33, Val64, Leu78, Phe80, Leu83, His84, Gln85, Leu134, Ala144, Asp145, Phe146, Leu148
5b	−52.51	−7.401	−70.033	—	—	Ile10, Gly11, Glu12, Gly13, Val18, Ala31, Lys33, Val64, Phe80, Glu81, Phe82, Leu83, Asp86, Gln131, Asn132, Leu134, Ala144, Asp145, Leu148
5c	−46.85	−6.143	−55.564	—	—	Ile10, Gly11, Gly13, Thr14, Val18, Ala31, Lys33, Val64, Phe80, Glu81, Phe82, Leu83, His84, Gln85, Ala144, Asp145, Leu148
5 d	−36.49	−6.579	−53.501	1‐Bond (O‐Leu83)	1‐Bond (Aromatic ring‐Phe80)	Ile10, Val18, Ala31, Lys33, Val64, Phe80, Glu81, Phe82, Leu83, His84, Gln85, Asp86, Leu134, Ala144, Asp145, Leu148
5e	−37.58	−5.144	−51.545	—	—	Ile10, Gly11, Glu12, Gly13, Val18, Ala31, Lys33, Val64, Phe80, Glu81, Phe82, Leu83, Asp86, Gln131, Asn132, Leu134, Ala144, Asp145

**Table 6:** Docking scores, binding free energies, and molecular interaction parameters of synthesized 3a–e and 5a–e compounds with ERK2 (PDB ID: 4QP2).

Compound	QPLD Score			Interactions			Amino acids
	MM‐GBSA (ΔG_bind_)	XPG/Docking Score	Glide Emodel	H‐bonds	Pi‐pi‐cation	Halogen Bonds	
3a	−31.74	−4.752	−60.443	2 Bonds (O‐Lys54, HN‐Met108)	1 Bond (Aromatic ring‐IMD402)	—	Ile31, Gly32, Glu33, Gly34, Val39, Leu107, Met108, Glu109, Lys114, Leu156, Asn158, Phe183, IMD402
3b	−30.67	−4.186	−56.684	2 Bonds (HN‐Ile31, HN‐Met108)	1 Bond (Aromatic ring‐IMD402)	2 Bonds (Cl‐Glu33, Cl‐Lys114)	Ser29, Tyr30, Ile31, Gly32, Glu33, Gly34, Val39, Ser41, Ala52, Lys54, Leu107, Met108, Glu109, Lys114, IMD402
3c	−33.88	−4.868	−57.917	1 Bond (HN‐Gly34)	—	—	Ile31, Gly32, Glu33, Gly34, Ala35, Val39, Ala52, Lys54, Ile84, Gln105, Asp106, Leu107, Met108, Lys151, Leu156, IMD402
3 d	−35.28	−7.764	−65.067	1 Bond (O‐Met108)	1 Bond (Aromatic ring‐IMD402)	—	Ile31, Gly32, Val39, Ser41, Arg50, Ala52, Lys54, Ile84, Asp106, Leu107, Met108, Glu109, Phe183, IMD402
3e	−30.66	−4.091	−59.429	1 Bond (HN‐Glu33)	1 Bond (Aromatic ring‐IMD402)	—	Ile31, Gly32, Glu33, Gly34, Ala35, Tyr36, Val39, Ala52, Lys54, Arg67, Met108, Glu109, Lys114, Lys151, Ser153, Asn154, Leu156, Asp167, IMD402
5a	−27.01	−4.121	−47.035	2 Bonds (HN‐Met108, HN‐Met108)	—	—	Ile31, Val39, Ala52, Lys54, Ile84, Gln105, Asp106, Leu107, Met108, Glu109, Lys114, Leu156, IMD402
5b	−35.62	−5.050	−61.993	1 Bond (HN‐Ile31)	1 Bond (Aromatic ring‐IMD402)	—	Ile31, Gly32, Val39, Ala52, Lys54, Ile84, Gln105, Asp106, Leu107, Met108, Glu109, Lys114, Leu156, IMD402
5c	−28.34	−4.953	−49.060	2 Bonds (HN‐Met108, HN‐Met108)	—	—	Ile31, Val39, Ala52, Lys54, Ile84, Gln105, Asp106, Leu107, Met108, Glu109, Lys114, Leu156, IMD402
5 d	−31.65	−4.869	−45.774	1 Bond (N‐Lys54)	2 Bonds (Aromatic ring‐Lys54, Aromatic ring‐IMD402)	—	Ile31, Gly32, Glu33, Gly34, Ala35, Val39, Lys54, Ile56, Tyr63, Glu71, Lys114, Asp167, IMD402
5e	−27.79	−4.030	−55.074	2 Bonds (N‐Glu71, N‐Asp167)	2 Bonds (Aromatic ring‐Lys54, Aromatic ring‐IMD402)	—	Ile31, Gly32, Glu33, Gly34, Ala35, Tyr36, Val39, Lys54, Ile56, Glu71, Lys114, Asp167, Gly169, IMD402

Similarly, compound 5b with CDK2 and ERK2 demonstrated a strong binding orientation with an MM‐GBSA energy of ‐52.51 and ‐35.62 kcal/mol, an XPG score of −7.401 and −5.050, and a Glide Emodel value of −70.033 and ‐61.993. The molecular interaction profile showed one strong hydrogen bond with HN‐Ile31 (ERK2). The interaction network revealed a prominent π–π stacking interaction between its aromatic ring‐IMD402, supported by van der Waals interactions with Ile10, Gly11, Glu12, Gly13, Val18, Ala31, Lys33, Val64, Phe80, Glu81, Phe82, Leu83, Asp86, Gln131, Asn132, Leu134, Ala144, Asp145, Leu148 (CDK2) and Ile31, Gly32, Val39, Ala52, Lys54, Ile84, Gln105, Asp106, Leu107, Met108, Glu109, Lys114, Leu156, IMD402 (ERK2). These results suggest that the designed derivatives, particularly 3 d and 5b, possess a strong affinity toward CDK2 and EKR2, indicating their potential as promising anti‐apoptotic modulators.

Docking analyses were further extended to the CDK2 and ERK2 proteins (PDB IDs: 1AQ1 and 4QP2, respectively) to explore the multitarget potential of the designed derivatives. The results revealed strong binding interactions across both targets, supported by favourable ΔGbind values, XPG scores, and MM‐GBSA energies (Tables 5 and 6). Compound 3 d again demonstrated superior stability, with MM‐GBSA energies of −51.74 kcal/mol (CDK2) and −35.28 kcal/mol (ERK2), XPG scores of −7.107 and −7.764, and Glide Emodel values of −72.329 and −65.067, respectively. In CDK2, compound 3 d established two hydrogen bonds with O–Lys33 and O–Asp145, while in ERK2, it formed one hydrogen bond with O–Met108, along with a π–π stacking interaction involving IMD402. Additional van der Waals contacts were identified with Ile10, Gly11, Glu12, Gly13, Val18, Ala31, Lys33, Val64, Phe80, Glu81, Phe82, Leu83, His84, Gln85, Asp86, Gln131, Asn132, Leu134, Ala144, Asp145, and Leu148 (CDK2), as well as Ile31, Gly32, Val39, Ser41, Arg50, Ala52, Lys54, Ile84, Asp106, Leu107, Met108, Glu109, and Phe183 (ERK2) (Tables 5 and 6 **)**.

Compound 5b also exhibited stable docking orientations with both CDK2 and ERK2, yielding MM‐GBSA energies of −52.51 and −35.62 kcal/mol, XPG scores of −7.401 and −5.050, and Glide Emodel values of −70.033 and −61.993, respectively. The interaction network revealed a hydrogen bond with HN–Ile31 (ERK2) and a π–π stacking interaction involving IMD402, supported by van der Waals interactions with Ile10, Gly11, Glu12, Gly13, Val18, Ala31, Lys33, Val64, Phe80, Glu81, Phe82, Leu83, Asp86, Gln131, Asn132, Leu134, Ala144, Asp145, Leu148 (CDK2) and Ile31, Gly32, Val39, Ala52, Lys54, Ile84, Gln105, Asp106, Leu107, Met108, Glu109, Lys114, Leu156, and IMD402 (ERK2) (Tables 5 and 6). Collectively, these molecular docking and MM‐GBSA results suggest that the designed derivatives, particularly 3 d and 5b, exhibit high binding affinity and stable interactions with Bcl‐2, CDK2, and ERK2 proteins. Their strong molecular interactions imply significant potential as lead scaffolds for the development of anti‐apoptotic and anticancer therapeutics.

### 3.9 Molecular Dynamics (MD) Simulations: Interaction Stability and Residue Fluctuation Analysis

Molecular dynamics (MD) simulations were conducted to evaluate the conformational stability and interaction dynamics of the 3 d and 5b ligand complexes with the Bcl‐2, CDK2, and ERK2 target proteins over a 150‐ns simulation period. Initially, all protein frames were aligned with the reference backbone structure, followed by the computation of the root mean square deviation (RMSD) for selected atoms to assess structural deviations. The ligand RMSD values were determined relative to both the protein and its respective binding site. The Bcl2–3 d, CDK2–3 d, and ERK2–3 d complexes demonstrated reduced RMSD values across multiple structural parameters, indicating high conformational stability throughout the simulation. The average RMSD values (in Å) for the α‐carbon (Cα), backbone, side chain, and all heavy atoms were recorded as 5.081, 6.031, and 2.769; 5.112, 6.275, and 2.765; 6.327, 7.201, and 4.015; and 5.740, 6.622, and 3.388, respectively (Figure 4a–c). Similarly, the Bcl2–5b, CDK2–5b, and ERK2–5b complexes exhibited favourable RMSD patterns, with the following values: Cα (3.295, 2.737, 4.770 Å), backbone (3.393, 2.759, 4.836 Å), side chain (4.840, 3.857, 5.398 Å), and all heavy atoms (4.116, 3.199, 5.092 Å), as illustrated in Figure 4d–f and summarized in S Table 2.

Root mean square fluctuation (RMSF) analyses revealed minimal residue‐level fluctuations in the 3 d and 5b complexes, further confirming stable ligand–protein interactions. The RMSF values (in Å) for 3d–Bcl2, 3d–CDK2, 3d–ERK2, and 5b–Bcl2, 5b–CDK2, 5b–ERK2 were as follows: Cα (4.679, 7.937, 6.494 and 5.314, 5.938, 6.265), backbone (5.259, 18.435, 6.957 and 5.839, 6.267, 6.745), side chain (6.156, 17.464, 6.684 and 5.503, 7.036, 6.492), and all heavy atoms (5.776, 18.044, 6.957 and 5.642, 6.570, 6.745) (Figure 5a–f and S Table 2). In addition to RMSD and RMSF, ligand descriptors such as radius of gyration (rGyr), intramolecular hydrogen bonds (intraHB), molecular surface area (MolSA), solvent‐accessible surface area (SASA), and polar surface area (PSA) were calculated to assess molecular compactness and solvation properties (Table 7). The SASA parameter, indicative of the solvent‐exposed surface critical for evaluating transfer free energy during solvation, remained within optimal limits, supporting consistent ligand accommodation within the binding pocket. Overall, both 3 d and 5b ligands demonstrated stable interactions with Bcl2, CDK2, and ERK2, as evidenced by favourable MD parameters: RMSD (2.53, 0.806, 2.714 and 0.91, 0.755, 0.774 Å), intra‐H bonds (0.0, 0.0, 0.0 and 0.0, 0.0, 1.0), rGyr (5.842, 4.621, 6.032 and 4.045, 4.177, 4.091 Å), PSA (234.75, 233.32, 237.97 and 35.49, 40.48, 30.09 Å), and SASA (131.80, 138.44, 377.68 and 98.85, 46.62, 76.77 Å). These results collectively indicate that compounds 3 d and 5b maintained strong and stable binding within the active sites of their respective targets throughout the simulation period.

**Table 7:** Comprehensive ligand dynamic properties derived from MD simulations of BCL2, CDK2 and ERK2 protein complexes with compounds 3 d and 5b.

Compound	RMSD	rGyr	IntraHB	MolSA	SASA	PSA
BCL2						
3 d	2.53	5.842	0	388.8	131.80	234.75
5b	0.91	4.145	1	268.79	98.85	40.48
CDK2						
3 d	0.806	4.621	0	387.84	138.44	233.32
5b	0.755	4.077	0	265.29	46.62	35.49
ERK2						
3 d	2.714	6.032	0	391.82	377.68	237.97
5b	0.744	4.091	1	266.37	76.77	30.09

Molecular dynamics simulations over 150 ns revealed stable interactions between the ligands and target proteins. Compound 3 d formed prominent hydrogen bonding, hydrophobic contacts, ionic interactions, and water bridges with key residues of Bcl2, CDK2, and ERK2. Similarly, 5b maintained consistent hydrophobic and hydrogen bond interactions, indicating stable binding within the active sites (Figure 6a‐f).

## 4 Discussion

The present investigation reports the successful synthesis, biological evaluation, and mechanistic validation of a novel series of fluorophenoxy‐picolinamide and aminobenzothiazole derivatives (3a‐e and 5a‐e) as multitarget anticancer candidates. The synthetic approach employed mild reaction conditions and afforded structurally diverse derivatives with good yields (Scheme 1 and S Table 1), confirming the versatility of the adopted methodology. Similar picolinamide and benzothiazole scaffolds have previously been reported to exhibit notable anticancer and antioxidant properties, supporting the rationale behind the molecular design of the present series [21, 22]. Oxidative stress plays a significant role in breast cancer progression, metastasis, and resistance to chemotherapy [23]. Therefore, the DPPH and ABTS assays were performed to evaluate the antioxidant potential of the synthesized compounds through two distinct radical‐scavenging mechanisms. DPPH primarily measures hydrogen atom donation, whereas ABTS assesses both hydrogen and electron transfer capacities in aqueous and organic systems. Using both assays provides a broader and more reliable assessment of antioxidant activity, which is relevant to anticancer potential because reduction of reactive oxygen species (ROS) can inhibit cancer cell proliferation and promote apoptosis. Thus, the findings from both assays support the potential role of these compounds in reducing oxidative stress‐mediated cancer progression. The Compounds 3 d and 5b demonstrated superior radical scavenging activity, with IC_50_ values closely comparable to ascorbic acid (Tables 1 and 2). The use of two complementary assays provides a broader assessment of antioxidant capacity. Notably, enhanced radical‐scavenging ability may contribute to modulation of oxidative stress–mediated cellular pathways, which are closely associated with cancer progression and apoptosis. Therefore, the observed antioxidant potential of compounds **3 d** and **5b** may partially correlate with their reported anticancer activity. These findings are consistent with earlier studies reporting that fluorinated aromatic systems and benzothiazole moieties enhance electron donating capacity and free radical stabilization [24]. The observed antioxidant activity may contribute to the cytoprotective modulation of intracellular redox balance, thereby sensitizing cancer cells to apoptotic stimuli.

The antiproliferative activity of the synthesized derivatives against triple‐negative MDA‐MB‐231 breast cancer cells revealed that compounds 3 d and 5b were the most potent, exhibiting IC_50_ values comparable to doxorubicin (Table 3). Importantly, fluorescence microscopy confirmed a marked reduction in viable cancer cells following treatment with these compounds (S Figure 6), while no cytotoxicity was observed in normal 3T3‐L1 cells (S Figure 6). This selective cytotoxicity is highly desirable and aligns with previous reports emphasizing the therapeutic advantage of benzothiazole‐based hybrids in minimizing off‐target toxicity [25]. Apoptosis analysis using Annexin V/PI staining demonstrated that 3 d and 5b significantly increased late apoptotic populations, comparable to doxorubicin (Figure 1). The induction of apoptosis rather than necrosis suggests activation of regulated cell death pathways, which is a hallmark of effective anticancer agents [26]. Consistently, cell‐cycle analysis revealed a pronounced G_2_/M phase arrest induced by these compounds (Figure 2a‐d). Arrest at the G_2_/M checkpoint is frequently associated with DNA damage and inhibition of cyclin‐dependent kinases, particularly CDK2, ultimately leading to mitotic catastrophe and apoptosis [27]. To further elucidate the molecular basis of these effects, qRT‐PCR analysis demonstrated significant down‐regulation of BCL2, CDK2, and ERK2 genes following treatment with compound 3 d (Figure 3). Overexpression of BCL‐2 is known to promote apoptosis resistance in breast cancer cells, while CDK2 and ERK2 play central roles in cell‐cycle progression and MAPK‐mediated proliferation, respectively [28, 29]. The coordinated suppression of these genes strongly supports the observed apoptotic induction and G_2_/M arrest, corroborating earlier mechanistic studies on multitarget anticancer agents [30].

Molecular docking studies further validated the experimental findings by demonstrating strong binding affinities of compounds 3 d and 5b toward Bcl‐2, CDK2, and ERK2 proteins (Tables 4, 5, 6 **)**. Compound 3 d, in particular, exhibited the most favourable MM‐GBSA energies and stable hydrogen bonding and π–π stacking interactions within the active sites (S Figures 7‐9). Such interactions are critical for effective inhibition of anti‐apoptotic and proliferative signaling pathways, as previously reported for small‐molecule inhibitors targeting these proteins [31]. The stability of these interactions was further confirmed through 150‐ns molecular dynamics simulations. Both **3d** and **5b** maintained stable binding conformations with minimal RMSD and RMSF fluctuations across all three protein targets (Figures 4 and 5; Table 7 and S Table 2). Favourable ligand descriptors, including radius of gyration and SASA values, indicate optimal ligand compactness and solvent accommodation, reinforcing the reliability of the docking predictions [19, 20]. The results suggest that compounds **3 d** and **5b** form stable interactions with Bcl2, CDK2, and ERK2 active site residues. Persistent hydrogen bonds, hydrophobic contacts, and water bridges indicate strong binding stability, supporting their potential inhibitory activity and validating the docking predictions (Figure 6). Structure‐activity relationship (SAR) analysis indicated that substitution on the picolinamide‐benzothiazole scaffold significantly influenced anticancer activity. Compounds bearing electron‐withdrawing groups, particularly in **3 d** and **5b**, enhanced binding interactions with BCL2, CDK2, and ERK2 active sites, improving cytotoxic efficacy. These findings are consistent with previously reported heterocyclic anticancer analogs, where fluorinated and benzothiazole moieties improved target affinity and biological activity [32, 33]. Overall, the integration of synthetic chemistry, antioxidant profiling, in vitro cytotoxicity, apoptosis and cell‐cycle studies, gene expression analysis, molecular docking, and MD simulations provides compelling evidence that compounds 3 d and 5b act as multitarget anticancer agents. Their ability to simultaneously modulate Bcl‐2‐mediated apoptosis resistance, CDK2‐driven cell‐cycle progression, and ERK2‐dependent proliferative signaling highlights their potential as promising lead scaffolds for further preclinical development against aggressive breast cancer subtypes.

## 5 Conclusion

The present study successfully designed, synthesized, and characterized two novel series of derivatives 4‐(4‐amino‐3‐fluorophenoxy)‐N‐methylpicolinamide (**3a–e**) and 2‐aminobenzothiazole (**5a–e**) through efficient synthetic routes. The structural integrity of all synthesized compounds was confirmed by FT‐IR, NMR, LC‐MS, and CHN analyses. Biological evaluations demonstrated that compounds **3d** and **5b** exhibited the most potent antioxidant and cytotoxic activities among the tested series. Both compounds displayed strong DPPH and ABTS radical scavenging capacities, comparable to ascorbic acid, along with significant cytotoxicity against MDA‐MB‐231 breast cancer cells while sparing normal 3T3‐L1 cells, suggesting high selectivity and therapeutic promise. Flow cytometric and fluorescence microscopy analyses revealed that **3d** and **5b** induced apoptosis and G2/M cell‐cycle arrest in MDA‐MB‐231 cells, consistent with qRT‐PCR data showing the down‐regulation of Bcl‐2, CDK2, and ERK2 gene expression. Molecular docking and MM‐GBSA analyses confirmed high binding affinities of **3d** and **5b** toward Bcl‐2, CDK2, and ERK2 proteins, supported by strong hydrogen bonding, π–π stacking, and van der Waals interactions. Furthermore, molecular dynamics simulations established the stability of ligand–protein complexes over 150 ns, affirming their robust interaction profiles. Overall, compounds **3 d** and **5b** emerge as promising lead scaffolds with dual antioxidant and anticancer potential, warranting further preclinical and mechanistic investigations.

## Author Contributions

**Nagalakshmamma Vadabingi:** formal analysis, investigation, **Venkataswamy Mallepogu**,**Surendra Pothuraju, Sumithra Poreddy**, **Chiranjeevi Pasala**, **Balaji Meriga:** formal analysis, investigation, **Nagalakshmamma Vadabingi**, **Venkataswamy Mallepogu**, **Balaji Meriga:** methodology, writing original draft, **Nagalakshmamma Vadabingi**, **Umamaheswari Amineni**, **Suresh Reddy Cirandur**, **Balaji Meriga:** conceptualization, **Nagalakshmamma Vadabingi**, **Venkataswamy Mallepogu**, **Balaji Meriga:** writingreview and editing, **Balaji Meriga:** supervision. All authors have read and agreed to the publishedversion of the manuscript.

## Funding

The authors have nothing to report.

## Conflicts of Interest

The authors declare no conflicts of interest.

## Supporting information


Supporting File

## Data Availability

The data that support the findings of this study are available from the corresponding author upon reasonable request.
