# Supplementary material for: Synthesis, Anticancer Evaluation, mRNA Expression Analysis, and Molecular Docking of Fluorinated Picolinamide–Benzothiazole Derivatives Against Breast Cancer‐Related Genes CDK2, ERK2, and BCL2
Source: J Biochem Mol Toxicol. 2026 Aug 17;40(9):e71025. doi: 10.1002/jbt.71025 (PMC13480355; doi:10.1002/jbt.71025)
Supplement: Supplementary file 1 [file JBT-40-e71025-s001.docx]

**Supplementary Material**

**Synthesis, anticancer evaluation, mRNA expression analysis, and molecular docking of fluorinated Picolinamide–Benzothiazole derivatives against breast cancer-related genes CDK2, ERK2, and BCL2**

**1 | Physical data of the newly 3(a–e) and 5(a–e) synthesized compounds**

The physical characteristics of the newly synthesized compounds, including 4-(4-amino-3-fluorophenoxy)-N-methylpicolinamide and 2-aminobenzothiazole derivatives, are presented in **S Table 1** of the supplementary data.

**2 | Proton Nuclear Magnetic Resonance (¹H NMR) Spectral Analysis**

The ¹H NMR spectra of compounds 3(a–e) and 5(a–e) were recorded in DMSO-d₆ and are consistent with the proposed molecular structures. The amide and thiourea –NH protons appear as singlets or broad signals in the downfield region (δ 8.0–11.6 ppm). The N-methyl protons of the picolinamide moiety resonate as doublets in the region of δ 1.9–2.9 ppm. Aromatic protons from the picolinamide, phenyl, and benzothiazole rings appear as multiplets in the range of δ 6.6–8.4 ppm. Representative ¹H NMR spectra of compounds **3(a–e)** and **5(a–e)** are presented in **S Figure 1a-e** and **S Figure 1f-j**.

**3 | Carbon-13 Nuclear Magnetic Resonance (¹³C NMR) Spectral Analysis**

The ¹³C NMR spectra of compounds 3(a–e) and 5(a–e) further support the assigned structures. Signals corresponding to aromatic carbons were observed in the region of δ 110–155 ppm, while the amide carbonyl carbon (C=O) resonated downfield at approximately δ 164–167 ppm. Thiocarbonyl and sulfonyl-substituted carbons appeared at their characteristic chemical shift values. All remaining aliphatic and heteroaromatic carbons appeared within their expected regions. Representative ¹³C NMR spectra of compounds **3(a–e)** and **5(a–e)** are presented in **S Figure 2a-e** and **S Figure 2f-j**.

**4 | Infrared (FT-IR) Spectral Analysis**

Fourier transform infrared (FT-IR) spectroscopy was employed to confirm the presence of key functional groups in the synthesized fluorinated picolinamide derivatives 3(a-e) and benzothiazole derivatives 5(a-e). The characteristic absorption bands observed around 3400–3920 cm⁻¹ correspond to **-**NH stretching vibrations, while bands in the region of 2930–2350 cm⁻¹ are attributed to aromatic C–H stretching. The strong absorptions appearing near 1650–1665 cm⁻¹ are assigned to the amide (C=O) stretching vibrations, confirming successful amide bond formation. Additional bands corresponding to sulfonamide, thiourea, and aromatic functionalities were observed in their expected regions. Representative FT-IR spectra of compounds **3d** and **5b** are provided in **S Figure 3a** and **S Figure 3b**.

**5 | High-Resolution Mass Spectrometry (HRMS)**

High-resolution mass spectrometric analysis was carried out in positive ionization mode for the synthesized compounds. The observed [M+H]⁺ peaks for compounds 3(a–e) and 5(a–e) closely matched their calculated molecular masses, confirming the molecular compositions. Representative HRMS spectra of compounds **3d** and **5b** are shown in **S Figure 4a** and **S Figure 4b**.

**6 | Elemental (CHN) Analysis**

Elemental (CHN) analysis was performed to determine the percentage composition of carbon, hydrogen, and nitrogen in selected compounds. The experimentally obtained values for compounds **3d** and **5b** were in good agreement with the theoretically calculated values, further validating the purity and structural integrity of the synthesized molecules. The elemental analysis reports are provided in **S Figure 5a** and **S Figure 5b**.

**7 |** **Cytotoxicity by MTT Assay**

The cytotoxic effects of the synthesized compounds (3a–e and 5a–e) were assessed against non-cancerous 3T3-L1 mammalian cells using the MTT assay at different concentrations (5, 10, 20, 30, 40, and 50 μg/mL) following 24 h of exposure. The obtained results are presented in **S Table 2**. Notably, all tested derivatives demonstrated negligible cytotoxicity toward 3T3-L1 mammalian cells across the tested concentration range, indicating their favorable safety profile toward normal cells.

**8 |** **Cell Viability by Microscopic Assay**

Fluorescence microscopic analysis presented in the Supplementary Data (**S Figure 6A–D**) demonstrated that treatment with compounds 3d and 5b significantly reduced the viability of MDA-MB-231 cells compared with untreated control cells (**S Figure 6A**). The observed cytotoxic effects (**S Figure 6B and C**) were comparable to those produced by the standard drug doxorubicin (**S Figure 6D**). In contrast, Supplementary Data (**S Figure 6E–H**) revealed no evident cytotoxicity in 3T3-L1 noncancerous mammalian cells following treatment with compounds 3d and 5b (**S Figure 6F and G**) or doxorubicin (**S Figure 6H**), thereby confirming the selective anticancer potential of these synthesized compounds.

**9 | RRD docking interaction analysis**

**S Figures 7–9** illustrate the rigid receptor docking (RRD) binding efficiency of synthesized compounds **3a–e** and **5a–e** with the target proteins **BCL2**, **CDK2**, and **ERK2**. The docking analysis demonstrates that several derivatives exhibited favourable binding affinities and stable interactions within the catalytic pockets. Notably, compounds **3d** and **5b** showed comparatively stronger docking scores and interaction patterns, indicating enhanced binding stability and potential inhibitory activity against these cancer-related targets.

**10 | Molecular Dynamics (MD) Simulations of RMSD and RMSF profiles**

**S Table 3** presents the RMSD and RMSF profiles obtained from 150 ns molecular dynamics simulations of BCL2, CDK2, and ERK2 complexes with compounds **3d** and **5b**. The relatively stable RMSD values and limited residue fluctuations indicate conformational stability of the protein–ligand complexes, supporting the strong binding interactions and structural integrity observed during the simulation period.

**S Table 1** Physical data of the title compounds.

| S.NO | Compound | Structure | Reaction Time  (hour) | Yield (٪) |
| --- | --- | --- | --- | --- |
| 1. | 3a |  | 5 | 90 |
| 2. | 3b |  | 5 | 93 |
| 3. | 3c |  | 5 | 92 |
| 4. | 3d |  | 5 | 94 |
| 5. | 3e |  | 5 | 93 |
| 6. | 5a |  | 5 | 95 |
| 7. | 5b |  | 5 | 95 |
| 8. | 5c |  | 5 | 93 |
| 9. | 5d |  | 5 | 95 |
| 10. | 5e |  | 5 | 94 |

**S Table 2** Effect of synthesized novel 3a-e and 5a-e title compounds on percentage of inhibition of 3T3 L1 cell viability by MTT assay

| Compounds | % of inhibition of 3T3 L1cells | | | | | | |
| --- | --- | --- | --- | --- | --- | --- | --- |
|  | 5 µg/mL | 10 µg/mL | 20 µg/mL | 30 µg/mL | 40 µg/mL | 50 µg/mL | IC_50_ µg/mL |
| 3a | 8.19 ± 1.1 | 8.29 ± 1.1 | 8.38 ± 1.1 | 8.41 ± 1.1 | 8.68 ± 1.2 | 8.96 ± 1.2 | 279.01 |
| 3b | 8.81 ± 1.1 | 8.91 ± 1.1 | 8.96± 1.2 | 9.02 ± 1.2 | 9.26 ± 1.2 | 9.39 ± 1.3 | 266.24 |
| 3c | 8.63 ± 1.1 | 8.75 ± 1.1 | 8.92 ± 1.2 | 9.0 ± 1.1 | 9.12 ± 1.2 | 9.30 ± 1.2 | 268.81 |
| 3d | 8.93 ± 1.1 | 8.96 ± 1.2 | 9.04 ± 1.2 | 9.18 ± 1.2 | 9.26 ± 1.2 | 9.29 ± 1.2 | 269.10 |
| 3e | 9.26 ± 1.1 | 9.37 ± 1.1 | 9.44 ± 1.2 | 9.51 ± 1.2 | 9.64 ± 1.2 | 9.88 ± 1.3 | 253.03 |
| 5a | 8.62 ± 1.1 | 8.69 ± 1.1 | 8.74 ± 1.1 | 8.88 ± 1.1 | 8.96 ± 1.1 | 9.02 ± 1.2 | 277.16 |
| 5b | 9.41 ± 1.1 | 9.50 ± 1.1 | 9.63 ± 1.2 | 9.81 ± 1.3 | 9.96 ± 1.3 | 10.02 ± 4.49 | 249.50 |
| 5c | 9.02 ± 1.1 | 9.15 ± 1.1 | 9.21 ± 1.1 | 9.28± 1.2 | 9.34 ± 1.2 | 9.39 ± 6.35 | 266.24 |
| 5d | 8.94 ± 1.1 | 8.99 ± 1.1 | 9.06± 1.1 | 9.14 ± 1.1 | 9.21 ± 1.2 | 9.28 ± 1.3 | 269.39 |
| 5e | 9.0 ± 1.1 | 9.06 ± 1.1 | 9.11 ± 1.2 | 9.19 ±1.2 | 9.23 ± 1.2 | 9.29 ± 1.2 | 269.10 |
| Doxorubicin | 9.44 ± 1.1 | 9.54 ± 1.1 | 9.68 ± 1.2 | 9.87 ± 1.2 | 10.10 ± 1.3 | 10.22 ± 4.49 | 244.61 |

**S Table 3** Protein RMSF and RMSD profiles of BCL2, CDK2 and ERK2 protein complexes with compounds 3d and 5b.

| **Compound** | **Alpha-C** | **Back bone** | **Side chain** | **All heavy** | **β-Factor** |
| --- | --- | --- | --- | --- | --- |
| P-RMSF-BCL2 | | | | | |
| 3d | 4.679 | 5.259 | 6.156 | 5.776 | 0.488 |
| 5b | 5.314 | 5.839 | 5.503 | 5.642 | 0.488 |
| P-RMSF-CDK2 | | | | | |
| 3d | 7.937 | 11.435 | 9.464 | 11.044 | 1.431 |
| 5b | 5.938 | 6.267 | 7.036 | 6.570 | 1.419 |
| P-RMSF-ERK2 | | | | | |
| 3d | 6.494 | 6.957 | 6.684 | 6.957 | 1.538 |
| 5b | 6.265 | 6.745 | 6.492 | 6.745 | 1.538 |
| PL-RMSD-BCL2 | | | | | |
| 3d | 5.081 | 5.112 | 6.327 | 5.740 | 0.522 |
| 5b | 3.295 | 3.393 | 4.840 | 4.166 | 0.412 |
| PL-RMSD-CDK2 | | | | | |
| 3d | 6.031 | 6.275 | 7.201 | 6.622 | 0.504 |
| 5b | 2.737 | 2.759 | 3.857 | 3.199 | 0.402 |
| PL-RMSD-ERK2 | | | | | |
| 3d | 2.769 | 2.765 | 4.015 | 3.388 | 0.411 |
| 5b | 4.770 | 4.836 | 5.398 | 5.092 | 0.458 |

**S Figure 1a (¹H NMR)**

**S Figure 1b (¹H NMR)**

**S Figure 1c (¹H NMR)**

**S Figure 1d (¹H NMR)**

**S Figure 1e (¹H NMR)**

**S Figure 1f (¹H NMR)**

**S Figure 1g (¹H NMR)**

**S Figure 1h (¹H NMR)**

**S Figure 1i (¹H NMR)**

**S Figure 1j (¹H NMR)**

**S Figure 2a (¹³C NMR)**

**S Figure 2b (¹³C NMR)**

**S Figure 2c (¹³C NMR)**

**S Figure 2d (¹³C NMR)**

**S Figure 2e (¹³C NMR)**

**S Figure 2f (¹³C NMR)**

**S Figure 2g (¹³C NMR)**

**S Figure 2h (¹³C NMR)**

**S Figure 2i (¹³C NMR)**

**S Figure 2j (¹³C NMR)**

**S Figure 3a** **(FT-IR)**

**S Figure 3b (FT-IR)**

**S Figure 4a** **(HRMS)**

**S Figure 4b** **(HRMS)**

**S Figure 5a** **(CHN)**

**S Figure 5b** **(CHN)**

**S Figure 6. Cell viability by microscopic assay:** Microscopic evaluation demonstrated that the newly synthesized compounds 3a–e and 5a–e exerted significant cytotoxic effects against **MDA-MB-231 cells.** (A) Untreated control cells, **(B) 3d-treated cells,** (C) 5b-treated cells, and **(D) doxorubicin-treated cells.** In contrast, no evident cytotoxicity was observed in **3T3-L1 noncancerous cells:** **(E) untreated cells,** (F) 3d-treated cells, (G) 5b-treated cells, and **(H) doxorubicin-treated cells**, confirming the selective anticancer activity of the synthesized compounds.

**S FIGURE 7** Binding efficiency results of **3a-e** and **5a-e** with BCL2 protein from rigid receptor docking (RRD) phenomena.

**S FIGURE 7 (Continued)**

**S FIGURE 8** Binding efficiency results of **3a-e** and **5a-e** with CDK2 protein from rigid receptor docking (RRD) phenomena.

**S Figure 8 (Continued)**

**S FIGURE 9** Binding efficiency results of **3a-e** and **5a-e** with ERK2 protein from rigid receptor docking (RRD) phenomena.

**S Figure 9 (Continued)**
